# Designing Values Elicitation Technologies for Mental Health and Chronic Care Integration: User-Centered Design Approach

**DOI:** 10.2196/68419

**Published:** 2025-02-19

**Authors:** Isabel R Rooper, William W Liem, Martha Burla, Jacob Gordon, Lara M Baez, Rachel Kornfield, Andrew B L Berry

**Affiliations:** 1 Department of Medical Social Sciences Northwestern University Feinberg School of Medicine Chicago, IL United States; 2 Center for Behavioral Intervention Technologies Northwestern University Feinberg School of Medicine Chicago, IL United States; 3 Institute for Sexual and Gender Minority Health and Wellbeing Northwestern University Feinberg School of Medicine Chicago, IL United States; 4 College of Nursing University of Cincinnati Cincinnati, OH United States; 5 Department of Preventive Medicine Northwestern University Feinberg School of Medicine Chicago, IL United States

**Keywords:** chronic care management, anxiety, depression, values elicitation, eHealth, patient-centered care, technology-enabled services, human-centered design, multimorbidity, user-centered design, multiple chronic conditions

## Abstract

**Background:**

Individuals with multiple chronic conditions (MCCs) and mental health challenges such as depression or anxiety have complex health needs and experience significant challenges with care coordination. Approaches to enhance care for patients with MCCs typically focus on eliciting patients’ values to identify and align treatment priorities across patients and providers. However, these efforts are often hindered by both systems- and patient-level barriers, which are exacerbated for patients with co-occurring mental health symptoms. Technology-enabled services (TES) offer a promising avenue to facilitate values elicitation and promote patient-centered care for these patients, though TES have not yet been tailored to their unique needs.

**Objective:**

This study aimed to identify design and implementation considerations for TES that facilitate values elicitation among patients with MCCs and depression or anxiety. We sought to understand the preferences of both clinicians and patients for TES that could bridge the gap between mental and physical health care.

**Methods:**

Using human-centered design methods, we conducted 7 co-design workshops with 18 participants, including primary care clinicians, mental health clinicians, and patients with MCCs and depression or anxiety. Participants were introduced to TES prototypes that used various formats (eg, worksheets and artificial intelligence chatbots) to elicit and communicate patients’ values. Prototypes were iteratively refined based on participant feedback. Data from these sessions were analyzed using reflexive thematic analysis to uncover themes related to service, technology, and implementation considerations.

**Results:**

Three primary themes were identified. (1) Service considerations: TES should help patients translate elicited values into actionable treatment plans and include low-burden, flexible activities to accommodate fluctuations in their mental health symptoms. Both patients and clinicians indicated that TES could be valuable for improving appointment preparation and patient-provider communication through interpersonal skill-building. (2) Technology considerations: Patients expressed openness to TES prototypes that used artificial intelligence, particularly those that provided concise summaries of appointment priorities. Visual aids and simplified language were highlighted as essential features to support accessibility for neurodiverse patients. (3) Implementation considerations: Clinicians and patients favored situating values elicitation in mental health care settings over primary care and preferred self-guided TES that patients could complete independently before appointments.

**Conclusions:**

Findings indicate that TES can address the unique needs of patients with MCCs and mental health challenges by facilitating values-based care. Key design considerations include ensuring TES flexibility to account for fluctuating mental health symptoms, facilitating skill-building for effective communication, and creating user-friendly technology interfaces. Future research should explore how TES can be integrated into health care settings to enhance care coordination and support patient-centered treatment planning. By aligning TES design with patient and clinician preferences, there is potential to bridge gaps in care for this complex patient population.

## Introduction

Approximately 42% of adults in the United States have multiple chronic conditions (MCCs), commonly including diabetes, heart disease, and hypertension [1]. Managing MCCs is a persistent challenge for both patients and their health care providers due to the complex health care needs of these patients. Care approaches that target one condition at a time have been deemed ineffective in addressing the complexities of competing health priorities [2,3], which leaves patients susceptible to gaps in care [4]. Specifically, recommended treatments and clinical workflows for each condition may conflict, causing patients to feel overwhelmed and unsupported in managing their complex health conditions. Patients with MCCs are also burdened with tracking and sharing medical information between providers, which complicates their ability to effectively communicate their health needs and priorities across their various care team members [5]. These challenges are intensified when patients’ chronic conditions include mental health challenges such as depression or anxiety. Symptoms such as amotivation, low mood, and concentration difficulties associated with depression or anxiety can impair patients’ decision-making capacity [6], making it challenging for them to identify and articulate their health priorities during values elicitation. These symptoms can lead to cognitive and emotional barriers, such as limited working memory capacity that can impair patients’ ability to reflect and communicate their values, which are critical steps for informed, patient-centered care planning [7] that centralizes what patients consider most important for their health and well-being [8,9]. The traditional separation between physical and mental health care providers further complicates coordinated care, exacerbating the challenges these patients face in aligning their care with their personal values [10].

Approaches such as the Patient Priorities Care model [11] have been developed to enhance care for patients with MCCs. Such approaches focus on identifying and aligning health priorities between patients and providers to better address their respective needs [12]. Common strategies include eliciting patients’ values to facilitate patient-provider discussions about priorities and inform patient-centered care management. Structured communication tools have shown promise in supporting patients with MCCs in incorporating their values into their treatment plans, including technology-enabled acceptance and commitment therapy and self-efficacy–enhancing interviewing techniques [13]. Despite this progress, current interventions still fail to meet the unique requirements of patients who also experience depression or anxiety. For instance, existing interventions do not adequately account for the ways in which mental health symptoms may impact patients’ capacity to engage with values elicitation activities [14]. Prior research in eliciting patients’ values among primary care physicians has also revealed significant communication barriers due to patient-level constraints (eg, overlapping symptoms, capacity to engage) and the perceived irrelevance of values in health care [15]. Structural barriers, such as time constraints and competing clinical priorities, have also led to low intervention uptake among providers [16], which signals the need for values elicitation interventions that are designed for implementation.

These barriers underscore the need for efficient interventions that (1) can be implemented within clinicians’ workflows, (2) are tailored to patients with MCCs and depression or anxiety, and (3) establish the relevance of values to health care. Recent work that explored the challenges faced by patients with MCCs and depression or anxiety in communicating their values to providers highlighted the need for user-friendly, flexible technology-enabled services (TES)—digital tools and platforms that enhance health care delivery by integrating technology into care—that facilitate values elicitation across both mental health and primary care settings [14]. Research is needed to further clarify patients’ and providers’ design preferences for values elicitation TES tailored to this population. Building on insights from past work focusing on patients’ preferences and needs in using TES for values elicitation [14], this study expands the scope to incorporate providers’ preferences and needs with respect to TES for values elicitation.

Human-centered design processes iteratively engage key parties (eg, patients and providers) through interactive methods such as co-design workshops to understand their goals, challenges, and motivations, and produce TES that adequately address key parties’ needs [17]. Given that implementation challenges have often limited the real-world impact of TES [18], implementation considerations should also be centered throughout design processes. The Accelerated Creation-to-Sustainment (ACTS) model [18] is a framework for expediting and improving the development and implementation of digital health solutions by integrating insights from human-computer interaction, implementation science, and clinical trial methodologies. The ACTS model aggregates design considerations into 3 groups: service, technology, and implementation. “Service” encompasses the behavioral strategies facilitated by digital tools and the expected roles of providers and patients; “technology” refers to the technologies that enable service delivery; and “implementation” involves methods to integrate digital tools into clinical practice, as well as broader contextual factors. This study used a human-centered design approach, informed by the ACTS model, to define design and implementation considerations for TES that help patients articulate their values and support providers in identifying and acting upon those values collaboratively.

## Methods

### Recruitment

Participants included both providers and patients. English-speaking providers with experience in primary care or mental health settings were recruited from a large medical-academic center in the Midwest United States via study advertisements and emails. Additional providers were reached through snowball sampling via personal contacts. Demographic information relevant to the foci of this study, such as role and department, was collected from providers. However, other demographic data were not gathered, as this analysis did not intend to assess demographic characteristics and their relationship to clinicians’ preferences regarding values elicitation TES. Patient participants were English-speaking adults with at least 2 self-reported chronic medical conditions and a history of depression or anxiety. The study prioritized engaging individuals with current or prior lived experience balancing mental and physical health challenges in order to capture nuanced insights into how these conditions intersect with chronic illness management and values elicitation. This approach aligns with human-centered design principles, emphasizing the value of incorporating a wide range of perspectives to inform participatory design [19]. Eligible patients were identified via institutional research recruitment registries and invited to participate in the study via email. Trained study personnel then scheduled interested individuals for workshops. Twelve providers and 6 patients verbally granted informed consent and participated in workshops.

### Ethical Considerations

This study was approved by the Northwestern University institutional review board (STU00212476). Informed consent was obtained from all participants prior to participation in the study. Participants were provided with detailed information about the study’s purpose, procedures, potential risks, and benefits, as well as assurances of confidentiality and the voluntary nature of their involvement. Participants were compensated with a US $50 gift card for their participation. Study data were deidentified to ensure participant privacy and confidentiality. Each participant was assigned a unique identification number for analysis and reporting purposes, and all personally identifying information was removed before sharing data outside the research team. Study data were securely stored in Health Insurance Portability and Accountability Act–compliant electronic locations.

### Workshops and Prototypes

#### Overview

Five provider workshops (workshops 1-5) and 2 patient workshops (workshops 6-7) were conducted via Zoom (Zoom Video Communications Inc) between February 2023 and March 2024 (Table 1). Researchers can identify themes and evaluate the most and least promising design directions with as few as 5-6 qualitative interviews [20]. Each workshop involved discussing and critiquing prototypes to explore specific aspects of TES design and implementation. Workshops lasted 60 minutes and included 4-6 participants (primary care providers [PCPs], mental health providers [MHPs], or patients) and 2 researchers (ABLB, JG, or WWL). At the beginning of each workshop, the researchers introduced themselves and established the workshop goals, confidentiality procedures, and prototype rationale. Prototypes shown in each workshop were tailored to address specific design questions, representing various values elicitation methods that were iteratively refined based on feedback from earlier workshops. Prototypes were intended as “thinking tools” to elicit design considerations, rather than high-fidelity prototypes to be optimized (see Table 2 for an overview of prototypes and Multimedia Appendix 1 for example prototypes) [14].

**Table 1:** Summary of workshop phases, objectives, activities, and prototypes used to explore clinicians’ and patients’ preferences for technology-enabled services for values elicitation, with prototypes tailored to specific goals in each phase.

Phase	Objectives	Activities	Prototypes	Purpose of prototypes
Phase 1 (N=4): Building buy-in for values elicitation (MHPs^a^ and PCPs^b^)	Explore how to communicate values elicitation rationale to patients.	Introduced rationale and workflow for values elicitation. Participants designed invitations to patients to engage in values elicitation.	Values bull’s eye worksheet [21]	Provide an example of a structured reflection tool on values and health priorities.
Phase 2 (N=4): Facilitating values elicitation (MHPs)	Understand how to facilitate discussions about patients’ values and their application in care planning.	Reviewed prototypes for eliciting and reflecting on values. Facilitated discussions on how to implement values elicitation in patient interactions. Drafted messages for MHPs to share values with PCPs.	Values bull’s eye worksheet	Provide an example of a structured reflection tool on values and health priorities.
			Premeeting with an MHP	Elicit reaction to completing a values exercise *before* meeting with an MHP.
			Story-sharing	Elicit reaction to completing a values exercise *while* meeting with an MHP.
Phase 3 (N=4): Shared care planning (PCPs)	Specify how patient values should be communicated to clinicians. Evaluate strategies and tools to enhance the effectiveness of shared care planning.	Critiqued patient scenarios and prototypes. Identified barriers to implementing shared care planning. Brainstormed templates and workflows for sharing values with PCPs.	Values bull’s eye worksheet	Provide an example of a structured reflection tool on values and health priorities.
			Inbox storyboard	Evaluate actionability of patients’ values in PCP visits.
			Previsit summary	Understand whether preappointment preparation could center patient values without adding significant workload burden.
			Workflow diagram	Illustrates care coordination between primary and mental health to explore proposed integrations.
Phase 3 (N=6): Shared care planning (patients)	Explore prototype methods to address challenges in identifying and communicating values, such as prioritizing health concerns, aligning values with treatment.	Explored patient-centered approaches for sharing values. Discussed patient feedback on interactive prototypes.	Story-sharing	Elicit reaction to completing a values exercise *while* meeting with an MHP.
			PCP simulator	Understand what types of communication-building support would be needed to communicate values to PCPs.

^a^MHPs: mental health providers.

^b^PCPs: primary care providers.

**Table 2:** Values elicitation technology–enabled service (TES) prototypes were shown to clinicians and patients to elicit their preferences for TES design and implementation^a^.

Workshop displayed	Prototype name and summary
W1-W3	Values bull’s eye worksheet [21]: Established tool from acceptance and commitment therapy to help patients reflect on their values via a mobile interface.
W2	Inbox storyboard: Workflow prototype in which patients complete values elicitation activities preappointment and mental health providers receive an inbox message with the results.
W4	Previsit summary: Document available in patients’ electronic health records listing patients’ personal values and appointment-related questions.
W4	Workflow diagram: Graphic displaying workflows to facilitate collaborative care management across mental and primary health care.
W5	Premeeting with an MHP^b^: Patients review self-care behaviors and assess their alignment with their values before meeting with an MHP; results electronically transmit to MHPs.
W5-W7	Story-sharing: Patients are emailed an exercise to complete preappointment and then meet with an MHP to reflect on their values and appointment priorities.
W6-W7	PCP^c^ simulator: Patients use an AI^d^ chatbot, playing the role of their PCP, to practice communicating their values to a dismissive PCP and receive takeaways (eg, communication tips and appointment priorities).

^a^The prototypes were used as “thinking tools” during the workshops.

^b^MHP: mental health provider.

^c^PCP: primary care provider.

^d^AI: artificial intelligence.

#### Prototype Development

Building on past work exploring the design requirements to support patients with MCC and anxiety or depression to identify and share their values [14], the team identified three key principles needed to support this population: (1) Credibility: the person eliciting the patient’s values has the training and experience to do so effectively and with beneficence. (2) Trustworthiness: sharing the patient’s values with the PCP will only benefit—and not negatively affect—the patient’s care. (3) Time worthiness: engaging in a reflection exercise to identify and communicate one’s values should offer meaningful benefits for their care, given competing demands on patients’ time and capacity (eg, appointments with multiple specialists).

**Figure 1:**
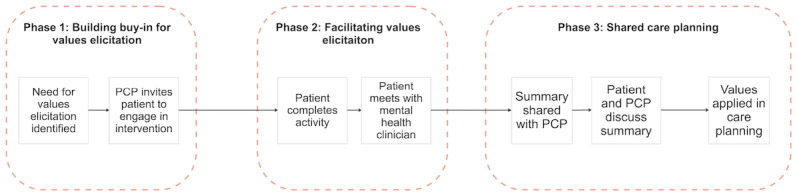
High-level overview of values elicitation service design, which guided the formulation of the technology-enabled prototypes that were shown to clinicians and patients as part of this qualitative study. PCP: primary care provider.

The team generated a high-level service flow illustrating how values could be elicited and then applied in shared care planning (Figure 1). The service flow included 3 phases for conducting values elicitation with MHPs and PCPs.

The first phase, building buy-in for values elicitation, focused on strategies to communicate the rationale for values elicitation to patients, as well as the credibility, trustworthiness, and timeworthiness of various approaches to values elicitation. Perspectives from both PCPs and MHPs were elicited to understand how to build trust and demonstrate the value of values elicitation from both physical and mental health care perspectives.

The second phase, facilitating values elicitation, aimed to refine the activities and technologies through which values could be elicited. Since previous research indicated that patients in this population often preferred engaging in values elicitation with MHPs [14], this phase centered on gathering MHPs’ perspectives on how to structure and instrument values elicitation activities.

Finally, the third phase, shared care planning, focused on applying elicited patient values in shared care planning between patients and PCPs, including possible implementation barriers and facilitators. PCPs’ insights were sought in this phase to identify workflow opportunities and challenges, given their central role in coordinating care across diverse clinical settings.

Prototypes were then developed to illustrate alternatives within and across these phases. For example, in phase 2, prototypes explored MHP-driven values elicitation versus patient-driven, self-guided values elicitation. By seeking confirmatory and refuting evidence from multiple participant groups in response to a range of possible design directions, this approach increased the rigor of the design research process.

#### Iterative Design Workshops

While workshops were being conducted, the research team met weekly to discuss and enumerate the insights garnered through each workshop, align them with the research questions and workshop objectives, and use these insights to guide prototype development for pursuant workshops. Accordingly, prototypes shown in later workshops responded to the input and critique from participants in earlier workshops.

Following the provider workshops, the research team refined 2 prototypes that were then presented to patients in 2 workshops to understand how their preferences aligned with providers’ preferences. These 2 prototypes integrated insights from provider workshops and previous research with this patient population [14], which found that patients were open to values elicitation activities facilitated by MHPs, as well as self-guided values elicitation. This prior work additionally found that while patients generally know what they want to discuss with providers, they often lack the communication skills to convey these priorities effectively. Accordingly, two prototype options were created, listed in Multimedia Appendix 1: (1) a story-sharing prototype with an MHP and (2) a “PCP simulator” focused on building patients’ communication skills. The artificial intelligence (AI) tool within the PCP simulator was well suited to this task, as it generated text tailored to different patient-provider scenarios, helping patients practice communicating their values and priorities with a range of provider responses.

### Analyses

This analysis combined 2 complementary approaches: iterative prototype analysis to refine specific aspects of a TES, and thematic analysis to generate insights across workshops. Together, these approaches provided a robust framework for exploring values elicitation and shared care planning in diverse health care settings. Grounded in reflection-in-action [22] and research through design [23], this dual focus allowed us to rigorously address the complexities of designing and implementing values-based health care.

#### Iterative Refinement of Tools

Throughout this iterative process, prototypes served as “thinking tools” to elicit participants’ feedback and address emerging questions about the design space. This approach aligns with research through design methodologies, where artifacts are both products of research and vehicles for generating insights. Prototype analysis was informed by reflection-in-action principles, emphasizing adaptive learning and real-time evaluation. Each phase of the workshops was analyzed iteratively to refine prototypes and activities.

For example, feedback was gathered on initial concepts to identify areas where tools could better address user needs. This feedback informed the iterative development of prototypes, with adjustments made to enhance their usability and relevance. Later, barriers to integrating prototypes into existing workflows were examined. Insights from this process guided further refinements to ensure that the tools aligned with practical implementation requirements.

#### Thematic Analysis

Workshops were recorded via Zoom and transcribed. Transcripts were then deidentified and edited for accuracy. Finalized transcripts were qualitatively analyzed for themes using reflexive thematic analysis [24,25] to synthesize insights across workshops. This analysis involved the following steps:

1. Codebook development: Lead coauthors IRR and WL reviewed transcripts and workshop insights to create an initial codebook. Five coders then applied this codebook to a sample transcript, refining it collaboratively to resolve discrepancies.
2. Coding and memoing: Coders independently applied the refined codebook to transcripts using Dedoose (SocioCultural Research Consultants LLC) and wrote memos to capture emerging themes.
3. Collaborative synthesis: Weekly team meetings facilitated the development of themes, which were subsequently grouped using the ACTS model into design considerations for service, technology, and implementation.

This thematic analysis triangulated stakeholder perspectives by engaging participants across clinical roles and settings to validate findings and highlight role-specific nuances. Analysts’ reflexivity, shaped by expertise in health education, technology design, and lived experience managing chronic conditions enhanced these findings. By combining iterative prototyping with reflexive thematic analysis, this methodology was intentionally designed to bridge theory and practice. Prototypes generated actionable insights for refining design elements, while thematic analysis captured the broader social and systemic contexts in which these tools would be implemented. This dual approach ensured that these findings were both grounded in stakeholder needs and positioned to contribute to future research and practice in values-based health care.

## Results

### Participants

From the primary care domain, participants included 4 physicians, 1 internal medicine physician’s assistant, and 1 nurse practitioner, hereafter referred to as “PCPs.” From the mental health domain, participants (“MHPs”) included 2 clinical psychologists, 3 behavioral care coordinators, and 1 medical social worker. This grouping reflects the participants’ primary practice contexts, with PCPs focused on managing physical health and chronic conditions, and MHPs primarily engaged in addressing mental health care needs. Full sample characteristics are shown in Tables 3 and 4.

**Table 3:** Sample characteristics of the clinicians who participated in workshops that explored the design and implementation of technology-enabled services for values elicitation.

ID	Domain	Role	Workshop
1	Primary care	Primary care physician	W1
2	Mental health	Patient care coordinator	W1
3	Mental health	Patient care coordinator	W1
4	Mental health	Patient care coordinator	W1
5	Primary care	Internal medicine physician assistant	W2
6	Primary care	Nurse practitioner	W2
7	Mental health	Psychiatrist	W3
8	Mental health	Medical social worker	W3
9	Primary care	Primary care physician	W4
10	Primary care	Primary care physician	W4
11	Mental health	Clinical psychologist	W5
12	Mental health	Clinical psychologist	W5

**Table 4:** Sample characteristics of the patients who participated in workshops that explored the design and implementation of technology-enabled services for values elicitation.

ID	Gender	Race	Hispanic	Age (years)	Chronic conditions	Workshop
13	Man	White	No	66	Cancer, glaucoma, and hyperlipidemia	W6
14	Woman	White	No	82	Arthritis, kidney or bladder problems, macular degeneration, and hypertension	W6
15	Woman	White	No	66	Hyperlipidemia, restless leg syndrome, IBS^a^, and arthritis	W6
18	Woman	White	No	48	Arthritis, asthma, cancer, and Hashimoto disease	W7
16	Man	African American	No	61	Diabetes, arthritis, hypertension, and chronic pain	W7
17	Woman	White	No	38	Diabetes, hyperlipidemia, asthma, autism spectrum disorder, and ADHD^b^	W7

^a^IBS: irritable bowel syndrome.

^b^ADHD: attention-deficit/hyperactivity disorder.

### Service Considerations

Service considerations reflect the unique experiences and goals of patients with MCCs and depression or anxiety. Patients and providers preferred service models for values elicitation that addressed concerns about anxiety, agenda setting, and time pressure during clinical visits. For instance, patients described how managing MCCs leads to anxiety before appointments with their PCP because they feel pressure to maximize their limited time together and had poor previous experiences with dismissive providers. To alleviate this anxiety, patients wanted support preparing for appointments. Specifically, patients said that values elicitation TES should produce a tangible outcome (eg, a list of questions) supporting preparedness and serving as memory aids. This goal reflected that their anxiety could interfere with their ability to remember information during appointments, as one participant described:

A tool like this [the PCP simulator] would help me, especially if I forgot to put some stuff in. It’ll make sure I don’t miss anything that I need to share with my primary care physician.

P16, Patient

Likewise, providers said that balancing MCCs makes agenda-setting challenging for some patients, so TES should be designed to help patients clarify their agenda for the appointment by reflecting on their health concerns and establishing priorities to address with providers.

Communication challenges were cited by providers (eg, due to patients’ mental health symptoms) and patients, who said that values elicitation TES could offer specific language to use during appointments, allowing patients to refer to pregenerated language instead of finding the right words in the moment, which can be challenging when experiencing anxiety. MHPs and patients said that TES that facilitate interpersonal skill-building (eg, practicing effective communication strategies) could improve preappointment anxiety and preparation, as well as provider-patient communication during appointments.

Participants reacted to TES prototypes using service models that were self-guided (ie, completed independently by a patient) versus provider-facilitated (ie, collaboratively completed by a patient and a facilitator). Both patients and providers preferred self-guided over facilitated TES due to appointment time constraints. They said that patients needed sufficient time and capacity to engage with values elicitation. Patients appreciated how self-guided TES could be completed on their own schedules.

Best case scenario, I would prepare about a week before a physician visit. My schedule means that may or may not happen. So, having the ability to do it at whatever time, but save that information would be incredibly important for me.

P18, Patient

In addition, patients described how it would be challenging for them to engage with values elicitation when their mental health symptoms are high, indicating that self-guided TES, provided well in advance of appointments, may address patients’ fluctuating needs and abilities.

My days are very different in that my level of anxiety and depression in some days dictate what I can and can’t do. So, on good days, [values elicitation] is something that I would definitely be interested in doing. But on not-so-good days, probably not so much.

P13, Patient

Providers agreed that, if facilitated, TES should be led by MHPs due to the relevance of values to mental health care and perceived constraints of PCPs, extrapolated in the section “Implementation Considerations.” One MHP said that in addition to mental health, clinicians facilitating values elicitation should understand *physical* health, in order to best support patients managing MCCs. They recalled an instance where lack of knowledge about a patient’s physical health condition was a barrier to their care:

The Crohn’s Disease was super important to improving the depression. But I had very little insight into what her treatment was for the Crohn’s Disease, [which] stunts my ability to work with her.

P12, MHP

Across these findings, patients and providers suggested a flexible service model that accommodates patient-directed or clinician-facilitated values elicitation and emphasized the need to support previsit communication skill-building.

### Technology Considerations

The majority of providers’ feedback focused on service considerations (“Service Considerations” section). In terms of technology, PCPs emphasized constraints of short appointment times and competing clinical priorities, meaning that any provider-facing TES interfaces must present concise, at-a-glance information about patients’ values to be acceptable. The primary requirement is to enable PCPs to identify and address the highest priority health issues in a limited time. When viewing the previsit summary prototype, one provider commented:

I would say there’s too much information there...Because remember, this guy has diabetes, but also is obese, and also...we need to talk about medication for the depression. So, I’m grateful I have a 40-minute appointment, but how can we address this in a 20-minute?

P9, PCP

PCPs also said that the interface should provide clear instructions for interpreting the “results” of values elicitation, such that those results could be applied to patient care.

Providers said that the technology should integrate into a patient’s electronic health record and facilitate information sharing between providers. For instance, an MHP said that they would not have time to translate a patient’s values into a message to the patient’s PCP, so the technology would need to facilitate that information-sharing process.

I don’t know that I would look at a screening tool, assess it, and then filter that information for the PCP. I wouldn’t have that time. So, if it’s available, I would think they can look at it or not.

P8, MHP

Patients were shown prototypes that differed in the inclusion of AI. Patients expressed varying levels of openness to the use of AI in values elicitation TES. Participants found the use of conversational AI for skill-building and appointment preparation (as in the “PCP simulator” prototype) helpful and were generally unconcerned with the AI delivery method, although 1 participant expressed mistrust of AI and said that they would prefer that a person facilitate the same process. Another participant described a lack of familiarity with the technology.

I have not had a lot of personal experience with AI. But conceptually, I’m not opposed to it. And I would not be opposed to it in this particular scenario.

P13, Patient

Across designs, patients expressed concerns that technologies enabling values elicitation must be accessible, particularly for neurodivergent patients and those with conditions such as depression and anxiety, which can affect cognitive processing and communication. For example, incorporating emoticons was suggested to simplify complex emotions and support users in expressing their values more easily. When asked whether they preferred the story-sharing prototype or the AI chatbot, all patient participants chose the “PCP simulator” for its skill-building and appointment preparation functionalities.

### Implementation Considerations

Providers said that values elicitation should be implemented in mental health care (rather than primary care) and preferred that patients use TES previsit to avoid wasting valuable appointment time. They identified specific barriers, unique to the primary care domain, that reduce the feasibility of implementing a values elicitation TES therein.

PCPs said that patients’ values were more relevant to MHPs than PCPs. Due to their training and focus on physical health, PCPs did not feel equipped or well-positioned to elicit patients’ values. Generally, PCPs also perceived values as nonactionable, meaning they did not view patients’ values as information they could apply in patient care. Accordingly, they preferred not seeing their patients’ values enumerated unless those values were explicitly connected to a patient’s primary physical health concern.

To me, it’s just too nebulous and complex for me to use that information...or know what it actually means. And how it relates to their current chief complaint. And how I’m going to take their value and their chief complaint and validate it in a way that I wouldn’t otherwise do.

P10, PCP

Compared with MHPs, who defined values in alignment with values elicitation literature (ie, what patients consider most important for their health and well-being [8,9]), PCPs conceptualized values in nonspecific and variable terms, at times conflating values with goals and social history.

I actually have done this [values elicitation] for the last 25 years; I’ve always been big on social history...“where did you go to high school?” Because immediately that opens up so many conversations and goes in different directions that you start to understand people’s values.

P1, PCP

While this comment shows the provider’s commitment to building relationships with patients, their misunderstanding of values elicitation complicates its implementation. It creates a false impression that PCPs are effectively identifying patients’ care priorities, when in fact, the potential of values elicitation to enhance patient care has not yet been fully realized.

Conflating values and goals similarly undermines the purpose of values elicitation, as goals are typically specific and shorter-term, while values represent the deeper, longer-term priorities guiding patients’ overall care decisions. For example, a goal might be “to lower blood pressure,” while the underlying value could be “to maintain independence and live an active life.” By focusing on clinical goals without addressing these broader values, providers risk delivering care that aligns with immediate clinical targets but neglects what truly matters to patients. As a result, patients may feel that their overall well-being is overlooked, compromising the effectiveness of patient priorities-aligned care [26].

Regarding implementation processes, providers highlighted the many moving pieces that must be accounted for when implementing TES within existing health care systems. For example, a PCP cited workflow integration concerns and said that it is difficult to implement requisite workflows to refer patients to TES and review resulting information when TES are new and infrequently used.

If it’s only one in 12 patients, the doctor’s workflow isn’t going to be used to that kind of a process. And it might be less accepted by the doctor.

P10, PCP

PCPs and MHPs also said that structural barriers, such as short appointments, meant that implementing a new TES during an appointment would be infeasible. However, some providers were open to reviewing patients’ values before an appointment, so long as the patients completed the TES independently outside their appointment time and the presentation of those values matched providers’ other requirements (eg, actionability).

## Discussion

### Principal Findings

This study used iterative prototyping of values elicitation TES to identify the needs and preferences of providers and patients managing multiple mental and physical health conditions. By applying the ACTS model, this study identified service, technology, and implementation considerations for TES design that help patients articulate their values and support providers in identifying and acting upon those values collaboratively. These findings largely supported the Patient Priorities Care approach [11], in that participants generally perceived identifying and aligning health priorities across clinicians and patients as useful. Findings also extended the Patient Priorities Care model to apply to patients with comorbid mental and physical health conditions. In particular, these patients’ mental health symptoms and related needs have not yet been well represented in values elicitation research. This population can benefit from clarifying and communicating their values to promote patient-centered care. Per these results, design recommendations are organized in accordance with the ACTS model and summarized in Table 5.

**Table 5:** Design recommendations for values elicitation technology–enabled services for patients with multiple chronic conditions and depression or anxiety, organized by the ACTS model’s 3 domains: service, technology, and implementation.

Design recommendation			Challenge addressed		Rationale
**Service**					
	Incorporate clear, structured reflection prompts that elicit values actionable for health treatment planning.	Difficulty translating elicited values into actionable health treatment plans.		Structured prompts help patients articulate and connect their values to their treatment planning, making their values actionable for providers.	
	Offer various services, including low-burden activities when mental health symptoms are high.	Mental health symptoms pose a barrier to engaging with values elicitation.		Providing a suite of tools can address patients’ diverse needs and allow flexibility when patients have low capacity for engagement.	
	Incorporate self-guided skill-building features to handle difficult conversations.	Difficulties with communication and preappointment anxiety.		Supports self-reflection and skill-building to enhance patient communication and manage preappointment anxiety.	
	When relevant, involve facilitators experienced in physical and mental health.	Lack of understanding between mental and physical health care.		Facilitators with joint expertise can holistically offer values elicitation to support patients with MCCs^a^.	
**Technology**					
	Create summaries of health priorities for patients to bring to appointments.	Patients experience anxiety and sometimes forget information during appointments.		Providing tangible outputs helps patients organize their priorities, reducing anxiety and helping avoid forgotten concerns.	
	Engage patients with intellectual disabilities in design work.	Intellectual disabilities can pose barriers to TES^b^ engagement.		Centering neurodiverse patients’ perspectives will improve tool accessibility.	
	When used, explain the clinical intent of TES that use AI^c^.	Some mistrust in the AI prototype.		Despite some mistrust, patients accepted the AI prototype due to its perceived use for skill-building.	
**Implementation**					
	Establish concordance among providers about how to conceptualize values.	Discordance poses barriers, as providers must understand a TES to implement it.		Aligning providers’ perceptions of the purpose and value of a TES will help facilitate TES implementation.	
	Design for real-world structural barriers (eg, communication constraints).	Lack of interprofessional communication infrastructure.		Designing for present care coordination challenges helps TES add value for patients and providers.	
	Center implementation considerations (eg, via the ACTS model) throughout TES design processes.	Difficulty fitting new TES into existing health care systems and workflows.		Engaging with implementers (eg, clinicians and staff) yields contextual considerations that could become implementation determinants.	

^a^MCCs: multiple chronic conditions.

^b^TES: technology-enabled services**.**

^c^AI: artificial intelligence.

### Service Considerations

Results indicate that TES should assist patients in preparing for difficult conversations during appointments and provide specific language to use during consultations, both of which help alleviate their preappointment anxiety. This study identified an opportunity to enhance Patient Priorities Care approaches by addressing a critical skill gap: participants expressed a need for support not only in generating values-aligned priorities but also in developing self-advocacy skills to communicate those priorities effectively during appointments. This finding highlights that TES outputs (eg, a list of questions) alone do not guarantee effective communication; rather, participants suggested that TES should actively support patient-provider communication by strengthening patients’ self-advocacy skills. This finding aligns with previous work showing that mental health symptoms can make patient-provider communication particularly difficult among patients with MCCs [27]. Yet, these findings contrast with earlier work that found that patients with MCCs and depression or anxiety preferred values elicitation facilitated by an MHP, given their credentials and the potential therapeutic benefits [14]. In this study, despite presenting an MHP-directed TES prototype, patients preferred a self-directed service model, which addressed their need to prepare for difficult conversations with providers during appointments by accounting for interpersonal dynamics with providers, including dismissiveness. This preference aligns with previous research indicating that interactions with dismissive PCPs can heighten anxiety and complicate communication about health-related values and priorities [28]. Participants may therefore have prioritized this issue over deeper therapeutic conversations about values with MHPs. Given that this patient population already experiences unique barriers to communicating their health priorities (eg, due to their overlapping symptoms), addressing these communication challenges through skill-building is a promising area for future design work and a strategy that can empower patients to engage more actively in their appointments, leading to better care coordination and collaboration [29].

The Serious Illness Conversation Guide (SICG) provides a patient-tested framework for addressing communication challenges and self-advocacy skill gaps [30], making it highly relevant for values elicitation. By emphasizing structured communication tools, the SICG guides clinicians through sensitive discussions about patient values, offering clear language and actionable steps to ensure that these conversations are patient-centered. This structured approach could inform the design of TES by incorporating tools that allow patients to practice articulating their values before appointments. Such features align with patients’ preference for self-directed models, enabling them to prepare for discussions in a low-pressure setting while retaining the SICG’s focus on clarity and structure. In addition, TES inspired by the SICG could include clinician training modules to improve active listening, equipping providers to better respond to patients’ expressed values. These combined strategies address key interpersonal dynamics, such as the need to counter dismissiveness, while empowering patients to effectively advocate for their priorities.

Findings illustrated the need for values elicitation service models to incorporate expertise from clinicians who understand the interplay between mental and physical health. Many MHPs lack access to PCPs’ medical notes that detail patients’ ongoing physical health issues, which are important for understanding how symptoms may overlap [31]. This gap places the burden on patients with MCCs to clearly articulate their complex health needs to both PCPs and MHPs, a task that is particularly challenging for those experiencing depression or anxiety. Literature suggests that patients with MCCs and mental health conditions often face cognitive and emotional barriers that make it difficult to convey the full picture of their health to providers [32]. This communication gap can hinder care coordination and increase the risk of fragmented or inadequate care. To address this, it is essential for TES to not only support values elicitation but also empower patients with tools that help them communicate their physical and mental health concerns across care teams and settings. Future research should explore these tools, particularly in promoting better care collaboration and interprofessional communication between providers in managing complex care needs.

### Technology Considerations

Results from this study underscore the diverse technology preferences among patients and providers, highlighting the need for a multifaceted approach to designing TES for values elicitation. Patients and providers emphasized that the technology interface must be concise and user-friendly, accommodating the limited time available during appointments. PCPs also said that they needed clear instructions for interpreting values elicitation results. These findings align with Patient Priorities Care approaches, which emphasize the need for values to be actionable to providers. Furthermore, these findings are consistent with literature showing that technology interfaces in health care must present clear, actionable information to be effective [33], as providers often face information overload, where the volume and complexity of available information exceed their capacity to process and use it [34]. In the context of values elicitation, interfaces that provide at-a-glance summaries of values and care priorities, and straightforward instructions for responding to those, can help alleviate the challenges of managing complex patient information within constrained appointment times. This finding aligns with design recommendations for eliciting patient values in clinical conversations, which emphasize that providers must be able to easily access and interpret patient values for effective care planning [35].

Patient feedback revealed that technology design must consider the cognitive and sensory needs of users, particularly those with mental health challenges or intellectual disabilities. Previous work found that cognitively and emotionally demanding values elicitation activities can be particularly challenging for individuals with depression or anxiety, suggesting that designs should balance requisite effort against potential benefits by making activities shorter, more enjoyable, and less taxing to encourage patient engagement [14]. These findings expand upon this to include accommodating diverse communication needs among patients with intellectual disabilities, as highlighted by suggestions to include visual aids such as emoticons in the interface. Existing literature on eHealth interventions for people with intellectual disabilities calls for more intentional participatory development and iteration with end users [36], emphasizing the importance of seeing the “whole picture” of both mental and physical health when designing values elicitation TES. As individuals age with MCCs, the complications of these conditions—combined with cognitive challenges such as depression, anxiety, and intellectual disabilities—may contribute to increased difficulty in engaging with health technologies. Future design work should therefore involve patients with intellectual disabilities and those managing MCCs to cocreate inclusive technologies that address the interconnected nature of physical and mental health, promoting accessibility for this historically underrepresented group.

While patients expressed general openness to using a generative AI tool to build communication skills, responses were mixed when it came to identifying and effectively communicating their values, particularly when dealing with dismissive providers. Some participants expressed uncertainty or mistrust of AI, preferring human facilitation. This hesitation is consistent with studies indicating that while AI has potential in health care, its acceptance is often tempered by concerns about trust and transparency [37,38], emphasizing the need for AI-based tools to be carefully designed and transparently integrated into patient care in order to build trust and acceptance [37]. This finding underscores the importance of clearly communicating the rationale and benefits of values elicitation TES, as patients prefer assurance about how their values will be used [14]. In the context of AI-based tools, transparency about the elicitation and application of these values becomes even more critical.

Given these varied preferences, a suite of tools may be indicated that address relevant behavioral targets (eg, appointment preparation and preappointment anxiety) using varied technology-based approaches (eg, skill-building, virtual worksheets, and meeting with a values elicitation facilitator). Future design work should continue mapping this array of preferences and iterate TES to accommodate them.

### Implementation Considerations

The approach used in this study to implement values elicitation TES reflects a broader perspective than typical Patient Priorities Care conceptions. Specifically, the research team sought to understand the various touchpoints that patients encounter across their care journeys, given their engagement with multiple providers across different settings. By expanding the perspective of inquiry beyond single conditions or discrete interactions, this study identified new design directions, particularly in understanding how to bridge the conceptualizations of values held by both patients and providers. Establishing a common understanding of values is essential to successfully implement values elicitation TES; otherwise, challenges may arise due to knowledge gaps regarding how and why to engage patients in TES for values elicitation. Future work is therefore needed to help PCPs identify the relevance of patients’ values to patients’ health, and potentially, to reframe values elicitation to more clearly resonate with their practice. Indeed, PCPs in this study highlighted the importance of linking patients’ values to patients’ health concerns to make those values actionable. This finding aligns with patients’ perspectives as well, in that patients may withhold values from providers that they perceive as irrelevant to their health care [15]. Because these varied conceptualizations of values and their perceived relevance to health care pose an implementation barrier, designing TES for values elicitation requires navigating and aligning these conceptualizations. Co-designing values elicitation educational materials with providers may help improve their perceptions of the relevance of values elicitation to this patient population, as well as prepare them to articulate its purpose to patients.

Furthermore, TES must reflect real-world challenges, such as the lack of care coordination infrastructure in many health care systems. Providers in this study said that values elicitation TES should integrate seamlessly with existing electronic health records and facilitate efficient information sharing between providers, which aligns with literature indicating the importance of interoperability and streamlined communication in improving care coordination. Yet, they also cited structural barriers that prevent interprofessional communication, which means that in the current system, patients must directly communicate their values to their PCP. Patient Priorities Care services—a common, evidence-based approach to values elicitation for patients with MCCs [11]—often assume that staff, such as medical assistants, are available and trained to support PCPs in this process. However, not all health systems are equipped with the resources or staffing models to integrate these roles effectively, limiting the feasibility of this approach in certain settings. In practice, the absence of such interprofessional communication infrastructure means that patients bear the burden of conveying their values themselves, a task made complicated when their mental health symptoms limit their ability to engage with values elicitation. The applicability of Patient Priorities Care tools to patients with MCCs and depression or anxiety could therefore be improved by accounting for these implementation challenges (eg, via self-guided instead of facilitated approaches that require staff resources), as well as this patient population’s unique service requirements (eg, tool responsiveness to high mental health symptoms).

Building on these challenges, the SICG emphasizes the need for system-level changes, such as integrating structured prompts into electronic health records and developing standardized documentation templates that allow clinicians to efficiently reference and act on patient values. By embedding values elicitation into routine workflows, these tools reduce reliance on additional staffing resources, making them more feasible for implementation across diverse health care settings. Furthermore, the SICG’s approach to training clinicians in communication strategies could be adapted to help providers articulate the relevance of values elicitation to patients and to foster greater patient engagement. These strategies align with findings from this study, particularly the need to link values to actionable health priorities and to ensure that TES design reflects real-world structural constraints. Incorporating these SICG-inspired interventions could enhance the scalability and effectiveness of TES by streamlining workflows, linking patient values to actionable health priorities, and addressing structural constraints across diverse care environments.

### Reflections on the ACTS Model

The ACTS model provided a vital framework for generating these findings, particularly in addressing the complex needs of patients with multiple mental and physical health conditions. By focusing on service, technology, and implementation, the model helped identify the design and implementation needs of values elicitation TES. It emphasized the importance of empowering patients in their health care interactions, creating user-friendly tools that accommodate diverse needs, and addressing the structural barriers that hinder care coordination. The ACTS model’s structured approach ensured that TES design is both practical and effective, facilitating seamless integration into the existing health care landscape, and ultimately, meeting the needs of this complex patient population.

### Limitations

Because this work principally engaged providers from a large medical-academic center, the implementation considerations identified (eg, lack of interprofessional communication and workflow integration concerns) may not apply to other clinical contexts. Per the ACTS model, future work should continue designing TES with institution-specific implementation considerations in mind, which other designers can identify by using similar methods to this study. In addition, the providers who participated in these workshops were interested in values elicitation; their preferences may therefore not be transferable to other contexts.

Due to the small sample size and highly iterative prototypes, consensus was not achieved regarding preferred TES design features or approaches. Despite this, findings offer distinct, novel, and significant insights to inform future TES design.

Sample characteristics pose another limitation. Demographic data were not collected from providers. Future work must collect providers’ demographics to address the potential influence of misalignments between patient and clinician identities on care delivery, as these differences may affect how values are perceived and prioritized in clinical interactions. In addition, most patient participants self-identified as White and non-Hispanic. The lack of racial and ethnic diversity among patients limits the generalizability of these results and must be addressed in subsequent research. Future studies would also benefit from assessing patients’ current levels of depression or anxiety to learn how symptom severity may shape patients’ preferences.

### Implications and Future Directions

This study found opportunities to address the unique needs of patients with MCCs and depression or anxiety by designing TES that account for patients’ fluctuating mental health symptoms, build patients’ communication skills, and facilitate care collaboration across mental and physical health care. Next steps from this study include conducting continued design work with providers, such as service journey mapping, to uncover additional barriers and facilitators to integrating values elicitation TES into real-world practice. Building on this engagement with patients and providers, future efforts should also expand to include stakeholders who shape systems-level decisions around adoption and implementation. Engaging hospital administrators, policymakers, and other system influencers can help address the structural barriers identified in this study, particularly in care coordination. This broader stakeholder involvement will be essential for integrating TES into health care systems effectively, ensuring that these tools are supported by the necessary resources and workflows.

### Conclusions

This study offers novel insights into the design and implementation of TES for values elicitation among patients with MCCs and depression or anxiety, who have been underrepresented in values elicitation research to date. Findings demonstrate a clear patient and provider preference for self-guided TES that support communication skill-building, enabling patients to articulate and advocate for their values, particularly when engaging with dismissive providers. This study extends prior values elicitation research by identifying and addressing important barriers faced by this patient population and offers design recommendations for other TES designers to apply these insights in practice. In addition, applying the ACTS model offered a novel approach that enabled the research team to identify and centralize implementation considerations. Indeed, this study underscores the practical challenges of integrating TES into existing health care workflows, highlighting the need for tools that work within time constraints and respond to structural barriers, such as limited care coordination resources. These insights provide a foundation for future TES development aimed at improving care coordination and patient-centered care for this complex patient population, emphasizing the importance of designing tools that are both scalable and sensitive to mental health symptoms. Future research should further explore co-design processes and strategies for overcoming real-world implementation challenges.

## Appendix

Multimedia Appendix 1 Workshop prototypes.

## Abbreviations

ACTS: Accelerated Creation-to-Sustainment
AI: artificial intelligence
MCCs: multiple chronic conditions
MHP: mental health provider
PCP: primary care provider
SICG: Serious Illness Conversation Guide
TES: technology-enabled service

## Data Availability

Deidentified data may be made available upon reasonable request to the corresponding author, provided that appropriate data use agreements are established to ensure the continued protection of participant confidentiality.

## References

[1] Benavidez GA, Zahnd WE, Hung P, Eberth JM (2024). Chronic disease prevalence in the US: sociodemographic and geographic variations by zip code tabulation area. Prev Chronic Dis.

[2] Institute of Medicine (2001). Crossing the Quality Chasm: A New Health System for the 21st Century.

[3] Redelmeier DA, Tan SH, Booth GL (1998). The treatment of unrelated disorders in patients with chronic medical diseases. N Engl J Med.

[4] Albreht T, Dyakova M, Schellevis FG, Van den Broucke S (2016). Many diseases, one model of care?. J Comorb.

[5] Murphy E, Doyle J, Hannigan C, Smith S, Kuiper J, Jacobs A, Hoogerwerf E, Desideri L, Fiordelmondo V, Maluccelli L, Brady A, Dinsmore J (2017). Perceptions and use of technology to support older adults with multimorbidity. Stud Health Technol Inform.

[6] Hindmarch T, Hotopf M, Owen GS (2013). Depression and decision-making capacity for treatment or research: a systematic review. BMC Med Ethics.

[7] Moran TP (2016). Anxiety and working memory capacity: a meta-analysis and narrative review. Psychol Bull.

[8] Friedman B, Hendry DG (2019). Value Sensitive Design: Shaping Technology With Moral Imagination.

[9] Lim CY, Berry ABL, Hirsch T, Hartzler AL, Wagner EH, Ludman EJ, Ralston JD (2017). Understanding what is most important to individuals with multiple chronic conditions: a qualitative study of patients' perspectives. J Gen Intern Med.

[10] Ee C, Lake J, Firth J, Hargraves F, de Manincor M, Meade T, Marx W, Sarris J (2020). An integrative collaborative care model for people with mental illness and physical comorbidities. Int J Ment Health Syst.

[11] Tinetti ME, Naik AD, Dindo L, Costello DM, Esterson J, Geda M, Rosen J, Hernandez-Bigos K, Smith CD, Ouellet GM, Kang G, Lee Y, Blaum C (2019). Association of patient priorities-aligned decision-making with patient outcomes and ambulatory health care burden among older adults with multiple chronic conditions: a nonrandomized clinical trial. JAMA Intern Med.

[12] Ongwere T, Cantor G, Clawson J, Shih P, Connelly K (2021). Design and care for discordant chronic comorbidities: a comparison of healthcare providers’ perspectives.

[13] Mosler F, Packer K, Jerome L, Bird V (2023). Structured communication methods for mental health consultations in primary care: a scoping review. BMC Prim Care.

[14] Liem WW, Lattie EG, Taple BJ, Stamatis CA, Gordon J, Kornfield R, Berry AB (2024). Improving collaborative management of multiple mental and physical health conditions: a qualitative inquiry into designing technology-enabled services for eliciting patients' values. Proc ACM Hum Comput Interact.

[15] Lim CY, Berry ABL, Hirsch T, Hartzler A, Wagner EH, Ludman E, Ralston JD (2016). 'It just seems outside my health': how patients with chronic conditions perceive communication boundaries with providers.

[16] Overbeck G, Davidsen AS, Kousgaard MB (2016). Enablers and barriers to implementing collaborative care for anxiety and depression: a systematic qualitative review. Implement Sci.

[17] Ratwani RM, Fairbanks RJ, Hettinger AZ, Benda NC (2015). Electronic health record usability: analysis of the user-centered design processes of eleven electronic health record vendors. J Am Med Inform Assoc.

[18] Mohr DC, Lyon AR, Lattie EG, Reddy M, Schueller SM (2017). Accelerating digital mental health research from early design and creation to successful implementation and sustainment. J Med Internet Res.

[19] Maguire M (2001). Methods to support human-centred design. Int J Hum Comput Stud.

[20] Guest G, Namey E, Chen M (2020). A simple method to assess and report thematic saturation in qualitative research. PLoS One.

[21] Lundgren T, Luoma JB, Dahl J, Strosahl K, Melin L (2012). The bull's-eye values survey: a psychometric evaluation. Cogn Behav Pract.

[22] Schön DA (1987). Educating the Reflective Practitioner: Toward a New Design for Teaching and Learning in the Professions.

[23] Zimmerman J, Forlizzi J, Evenson S (2007). Research through design as a method for interaction design research in HCI.

[24] Braun V, Clarke V (2006). Using thematic analysis in psychology. Qual Res Psychol.

[25] Braun V, Clarke V (2020). Can I use TA? Should I use TA? Should I not use TA? Comparing reflexive thematic analysis and other pattern-based qualitative analytic approaches. Couns Psychother Res.

[26] Tinetti ME, Hashmi A, Ng H, Doyle M, Goto T, Esterson J, Naik AD, Dindo L, Li F (2024). Patient priorities-aligned care for older adults with multiple conditions: a nonrandomized controlled trial. JAMA Netw Open.

[27] Choi BM, Obeng-Kusi M, Axon DR (2022). Association between patient-provider communication and self-perceived mental health in US adults with cancer: real-world evidence through medical expenditure panel survey. Diseases.

[28] Hildenbrand GM, Perrault EK, Rnoh RH (2022). Patients' perceptions of health care providers' dismissive communication. Health Promot Pract.

[29] Menear M, Dugas M, Careau E, Chouinard M, Dogba MJ, Gagnon M, Gervais M, Gilbert M, Houle J, Kates N, Knowles S, Martin N, Nease DE, Zomahoun HTV, Légaré F (2020). Strategies for engaging patients and families in collaborative care programs for depression and anxiety disorders: a systematic review. J Affect Disord.

[30] (2023). Serious illness conversation guide. Ariadne Labs.

[31] Torab-Miandoab A, Samad-Soltani T, Jodati A, Rezaei-Hachesu P (2023). Interoperability of heterogeneous health information systems: a systematic literature review. BMC Med Inform Decis Mak.

[32] Maizes V, Rakel D, Niemiec C (2009). Integrative medicine and patient-centered care. Explore (NY).

[33] AlQudah AA, Al-Emran M, Shaalan K (2021). Technology acceptance in healthcare: a systematic review. Appl Sci.

[34] Hall A, Walton G (2004). Information overload within the health care system: a literature review. Health Info Libr J.

[35] Berry A, Lim C, Hartzler A, Hirsch T, Ludman E, Wagner EH, Ralston JD (2017). Creating conditions for patients' values to emerge in clinical conversations: perspectives of health care team members. https://europepmc.org/abstract/MED/28890950.

[36] van Calis JFE, Bevelander KE, van der Cruijsen AWC, Leusink GL, Naaldenberg J (2023). Toward inclusive approaches in the design, development, and implementation of eHealth in the intellectual disability sector: scoping review. J Med Internet Res.

[37] Megaro A, Visvizi A, Troisi O, Grimaldi M (2023). Transparency in AI systems for value co-creation in healthcare. Big Data and Decision-Making: Applications and Uses in the Public and Private Sector. Emerald Studies in Politics and Technology.

[38] Panigutti C, Beretta A, Fadda D, Giannotti F, Pedreschi D, Perotti A, Rinzivillo S (2023). Co-design of human-centered, explainable AI for clinical decision support. ACM Trans Interact Intell Syst.

