# Supplementary material for: Designing Values Elicitation Technologies for Mental Health and Chronic Care Integration: User-Centered Design Approach
Source: JMIR Form Res. 2025 Feb 19;9:e68419. doi: 10.2196/68419 (PMC11887998; doi:10.2196/68419)

**Multimedia Appendix 1. Workshop prototypes.**

**Multimedia Appendix 1A.** Values bullseye worksheet (Workshops 1-3). As one of our workshop prototypes, we used an established tool from Acceptance and Commitment Therapy to help patients reflect on their values, originally created by Lundgren (2012) and colleagues.^21^ The worksheet prompts patients to reflect on what matters most to them (i.e., their values) and offers several potential value areas (e.g., work, relationships, personal growth, leisure) for patients to consider. Then, patients indicate the extent to which they are living in alignment with those values. For instance, if a patient is *not* living in line with their values, they would place an ‘X’ in the outermost ring only. By contrast, if they are living fully in line with their values, they would mark an ‘X’ in the centermost ring (i.e., the bullseye). In the prototype we showed participants, the worksheet is completed via a mobile interface. Below, we reproduce a similar version of the worksheet, using non-copyrighted material.

First, patients select which values are most important to them:

**
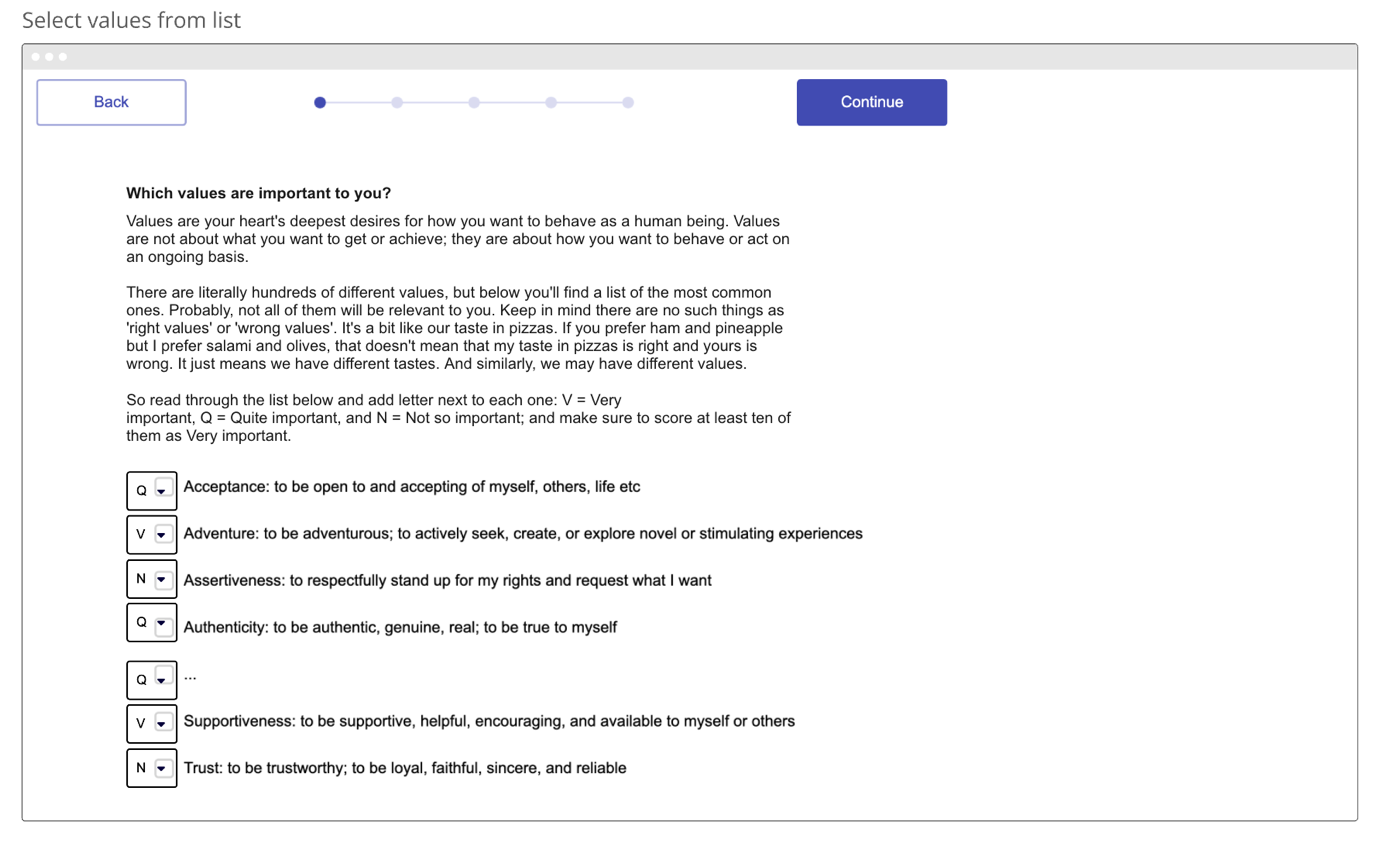
**

Then, they indicate the extent to which they are living in line with their values by filling in the appropriate ring. In the example below, the patient is living very much in line with their values of supportiveness and acceptance; somewhat in line with their values of trust and authenticity; and not at all in line with their values of assertiveness and skillfulness.

**
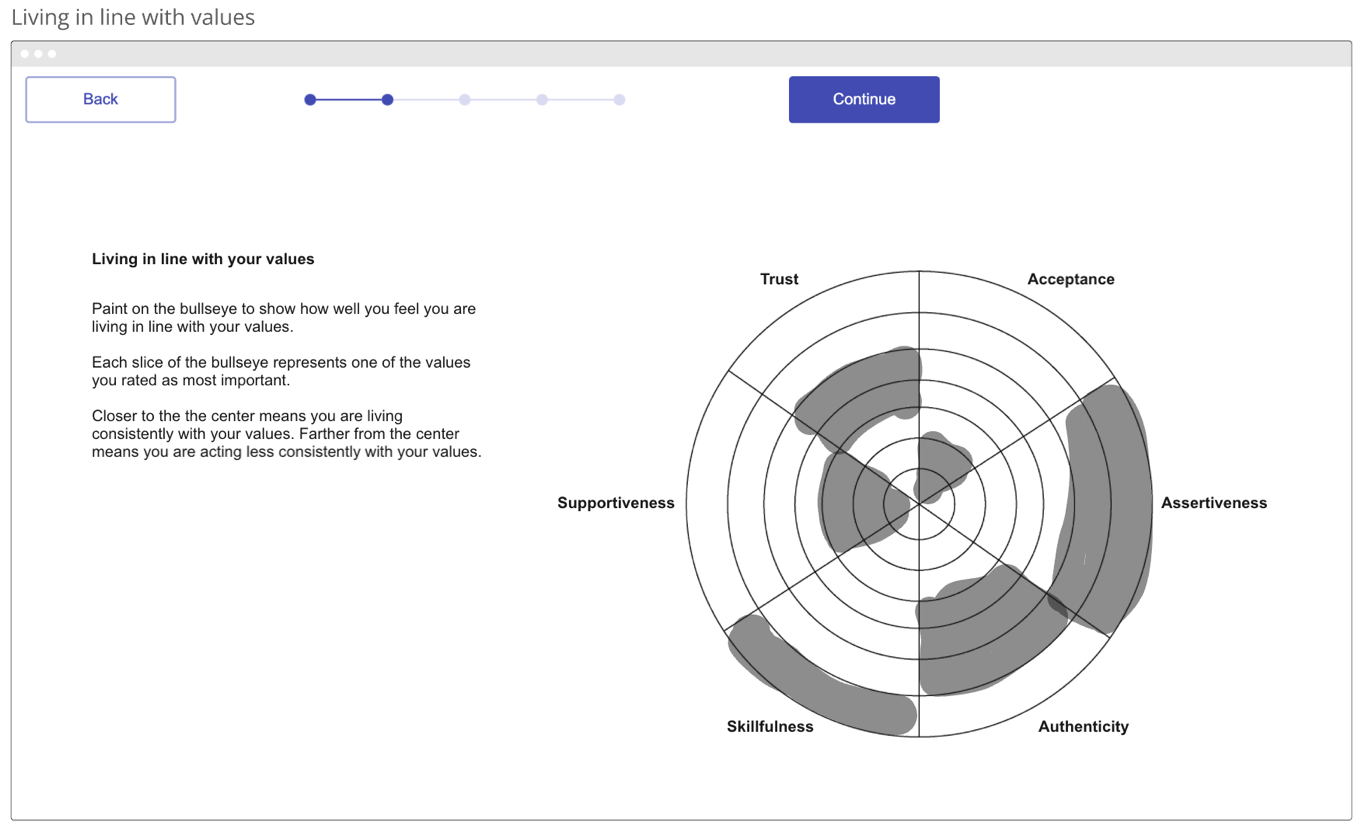
**

**Multimedia Appendix 1B.** Inbox storyboard (Workshop 2). Workflow prototype in which patients complete values elicitation pre-appointment and MHPs receive an inbox message displaying the results.

**
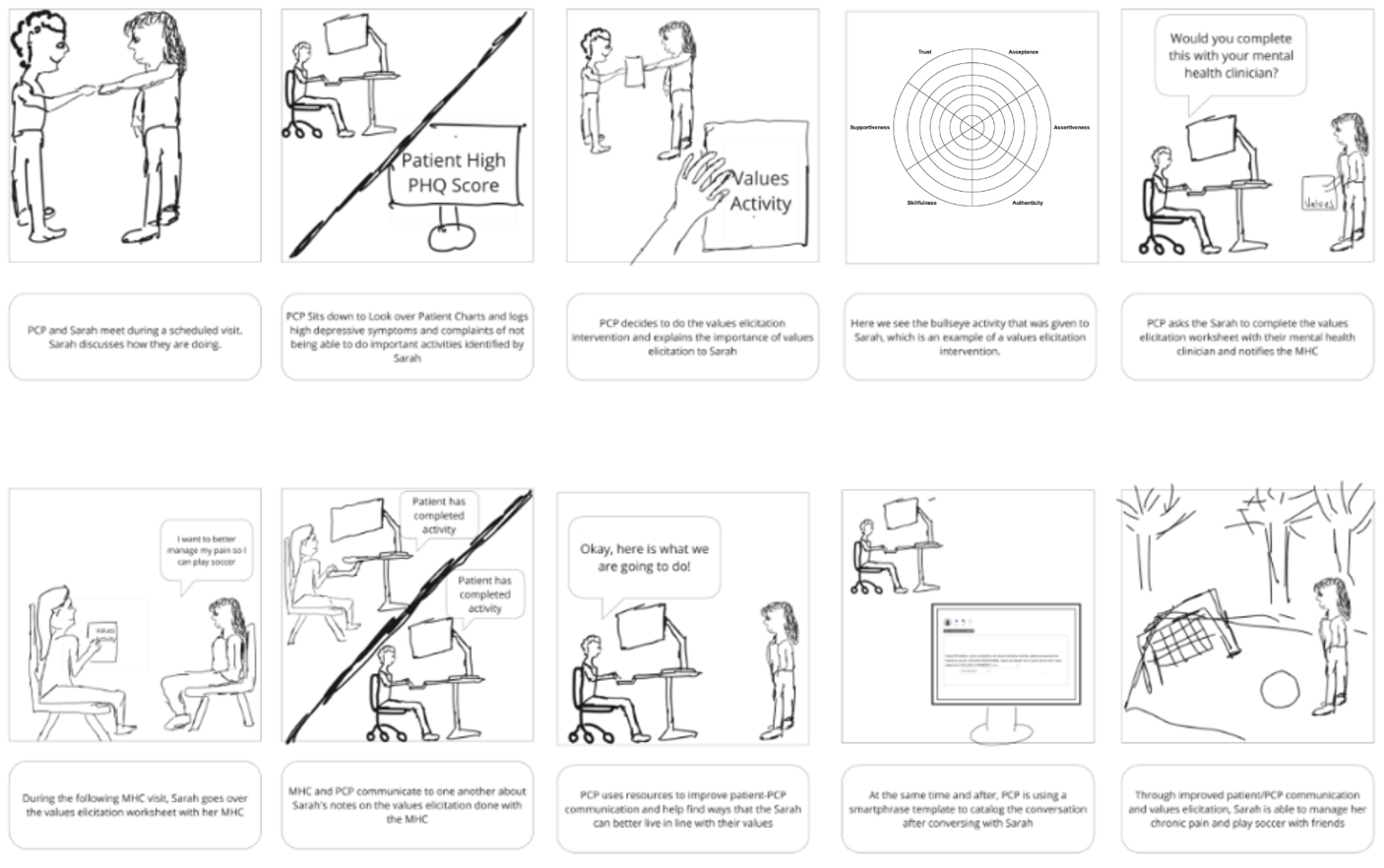
**

**Multimedia Appendix 1C.** Pre-visit summary (Workshop 4). Document available in patients’ electronic health records listing patients’ personal values and appointment-related questions.


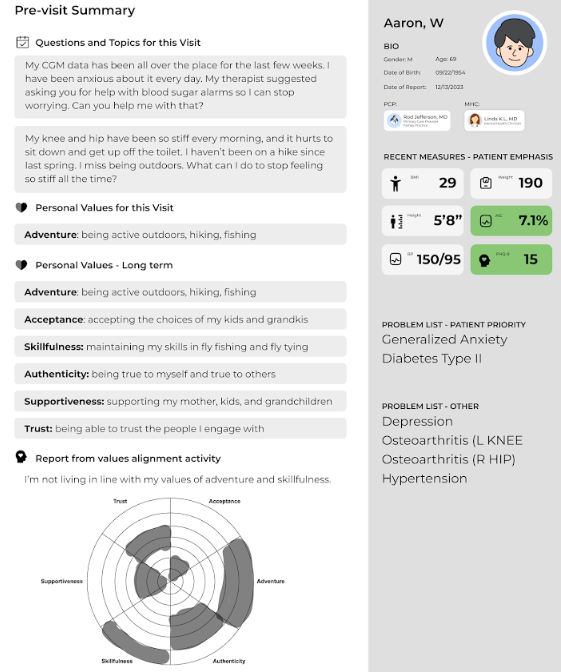


**Multimedia Appendix 1D.** Workflow diagram (Workshop 4). Graphic displaying workflows to facilitate collaborative care management across mental and primary health care.


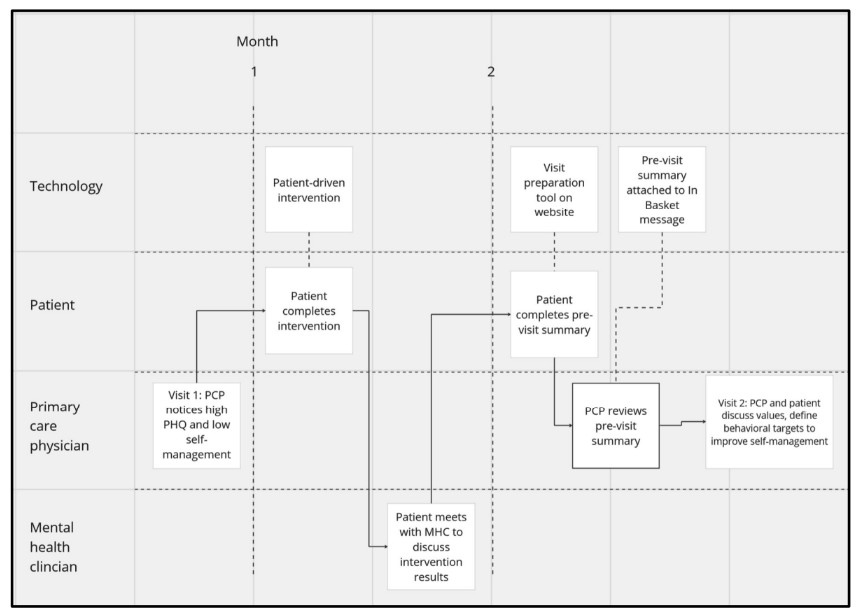


**Multimedia Appendix 1E.** Pre-meeting with an MHP (Workshop 5). Patients review self-care behaviors and assess their alignment with their values before meeting with an MHP; results are electronically transmitted to MHPs.

Patient sees a list of recommended self-managed health behaviors based on their unique multiple chronic condition’s intersection.

For each health behavior recommendation, the patient will argue why it will not work.

**Outcome:** Patient will have reflected on how particular self-care behaviors align or misalign with their skills and values.


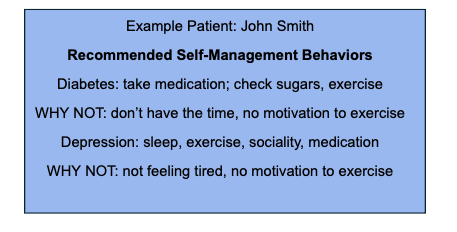


**Multimedia Appendix 1F.** “Story-sharing” (Workshops 5-7). Patients are emailed a reflective exercise to complete pre-appointment, then meet with an MHP to reflect on their values and appointment priorities.


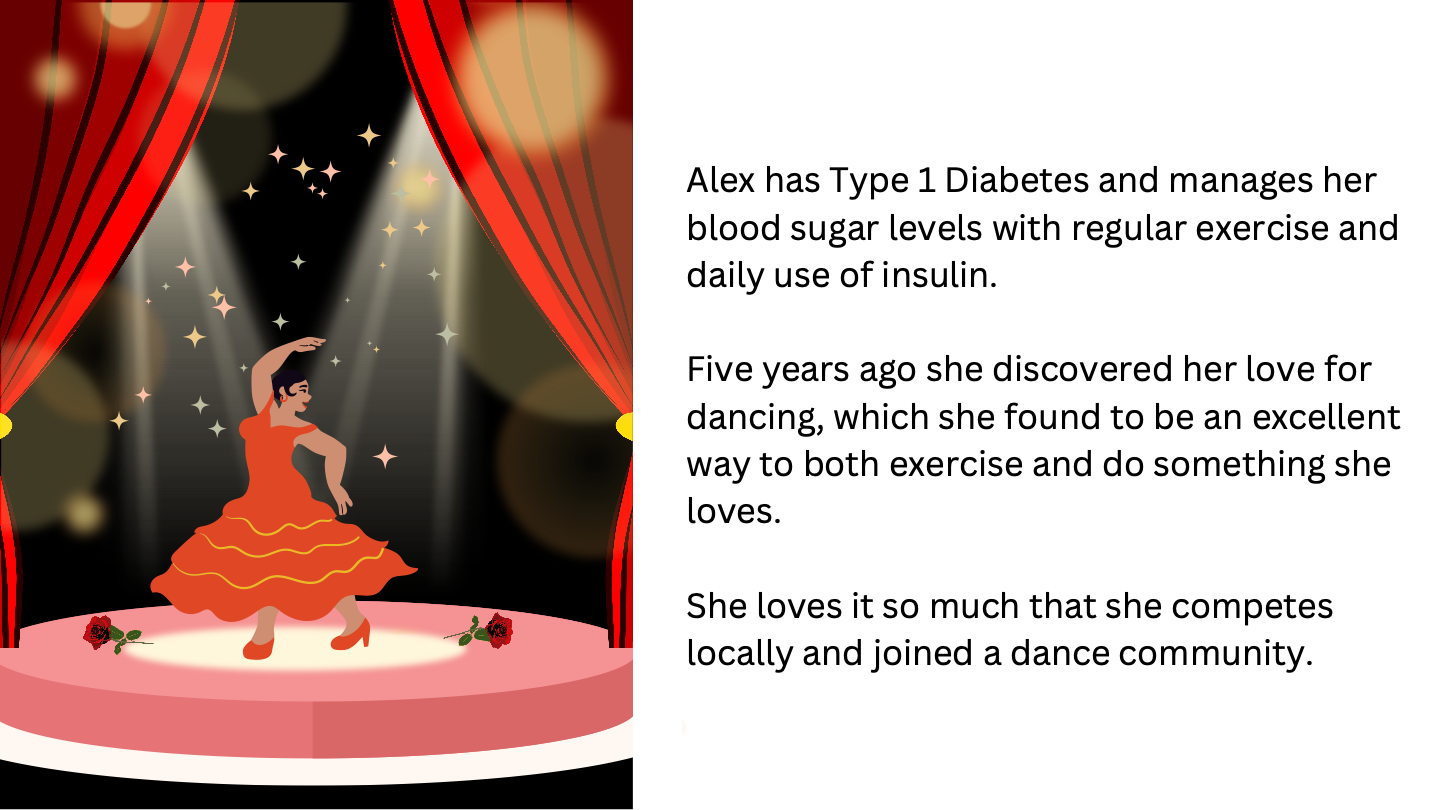


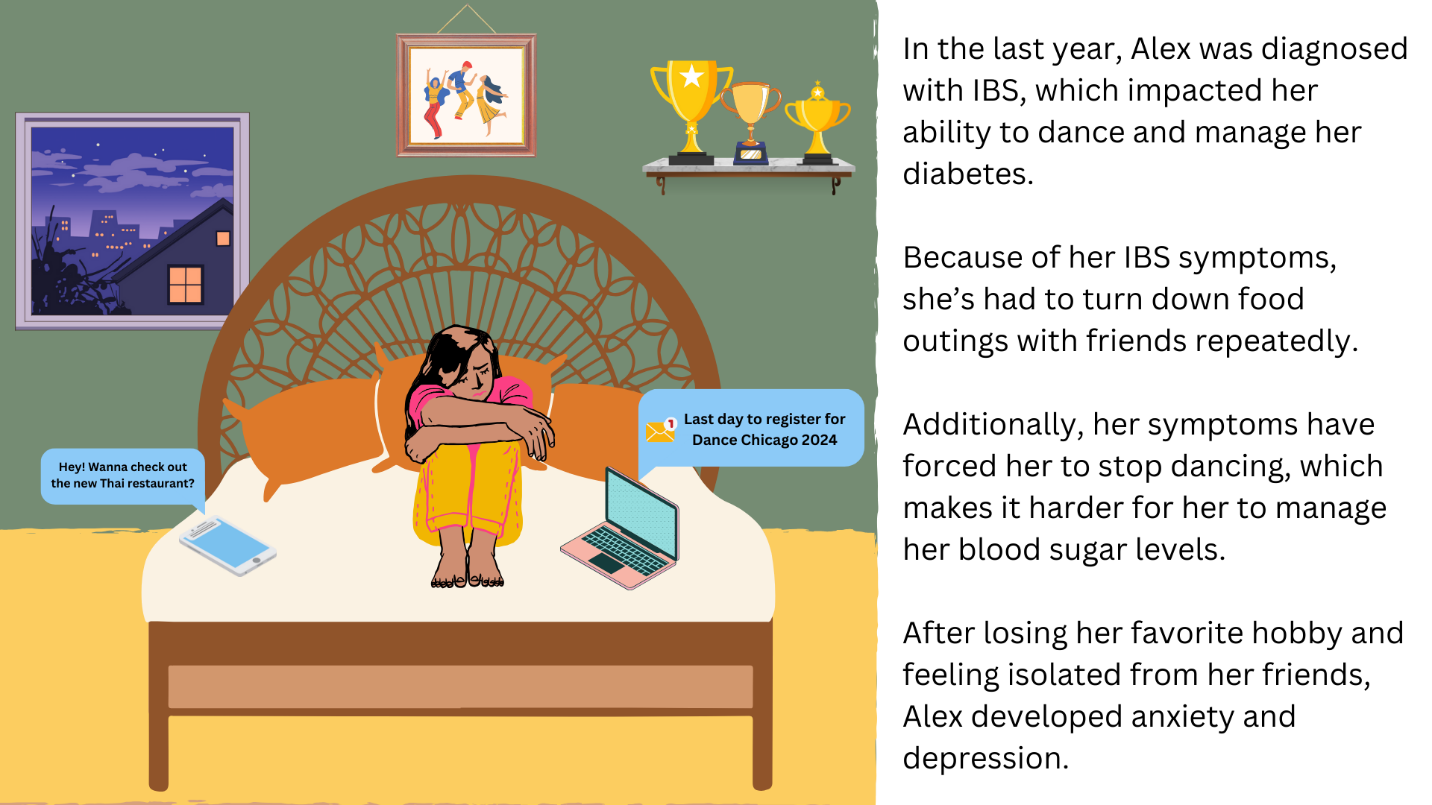


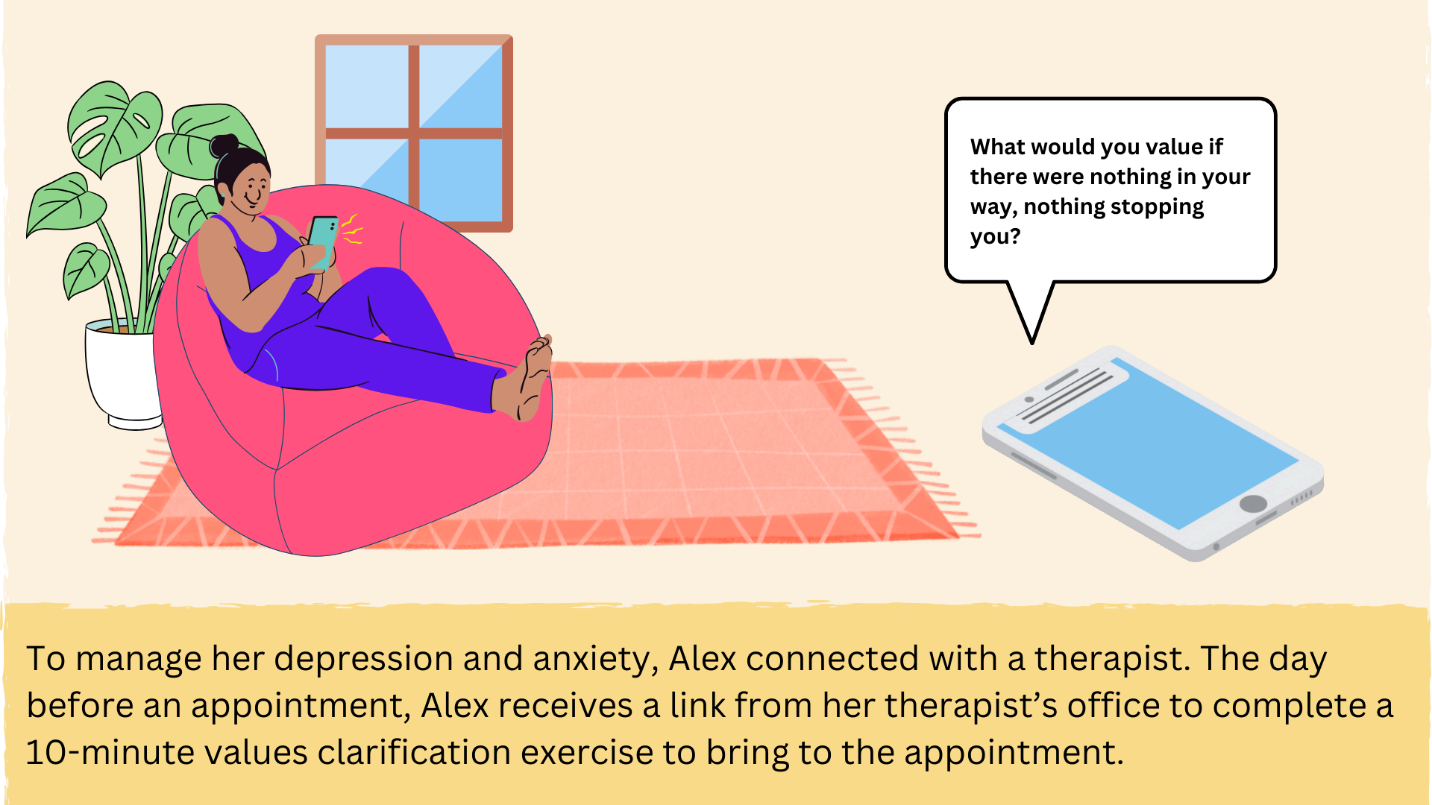


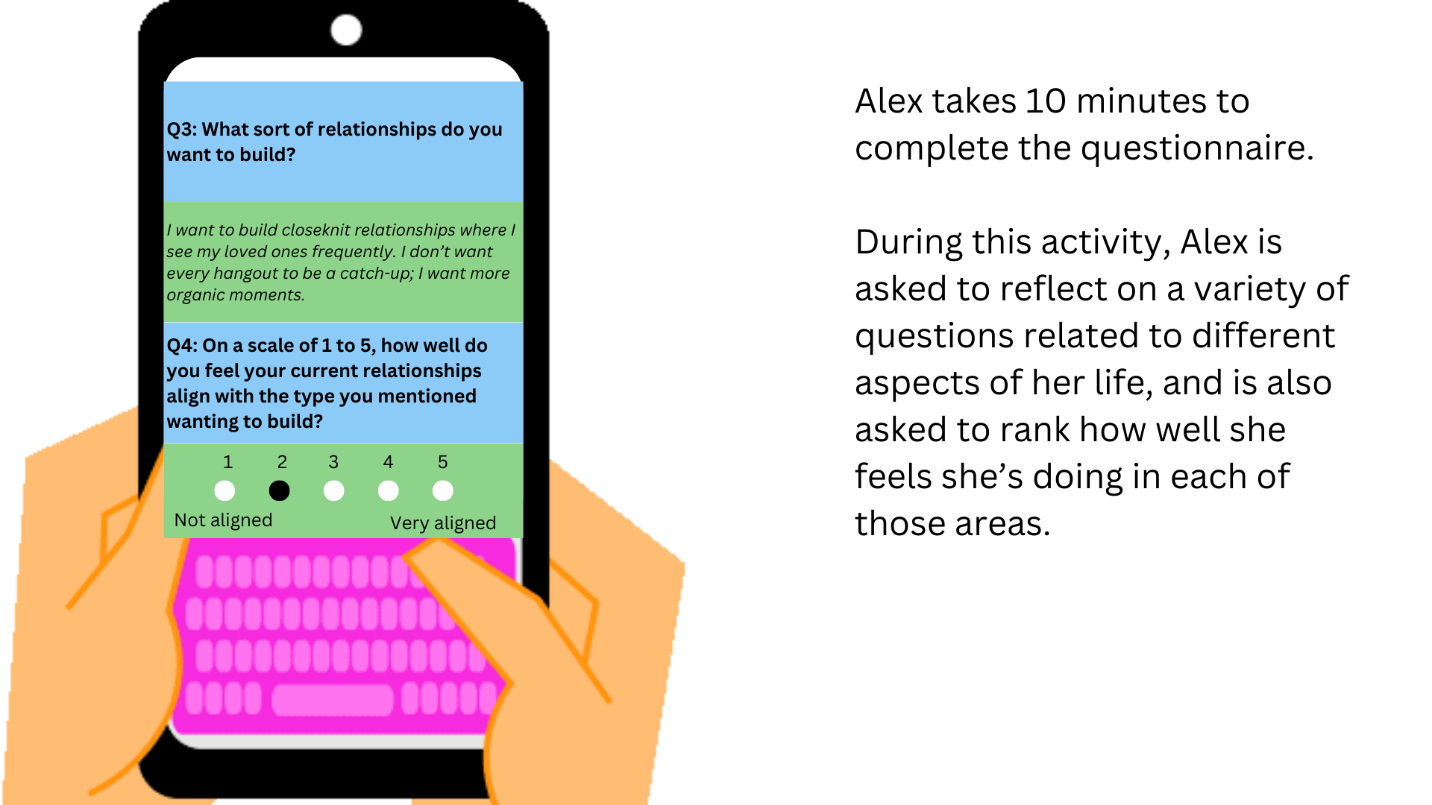


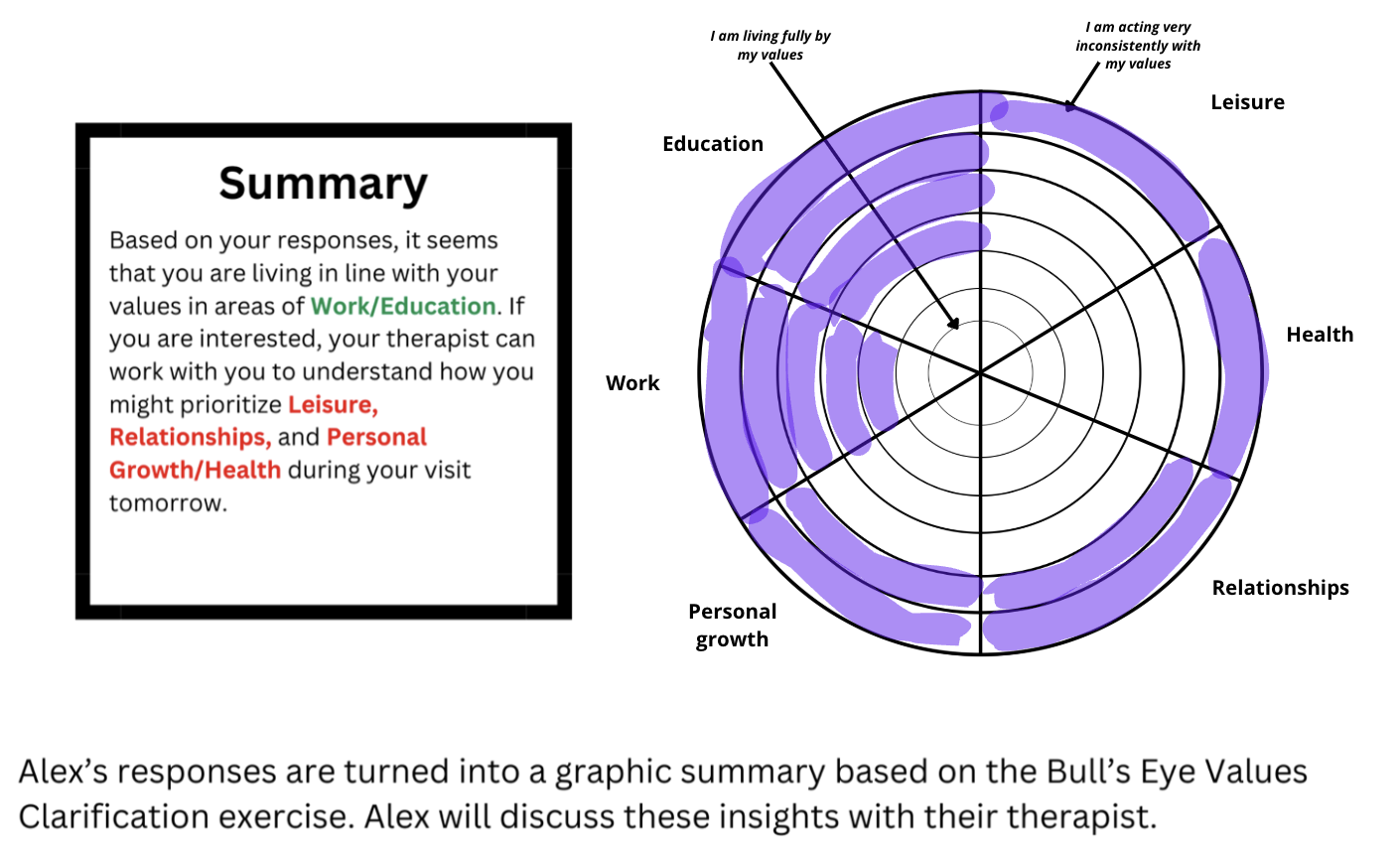


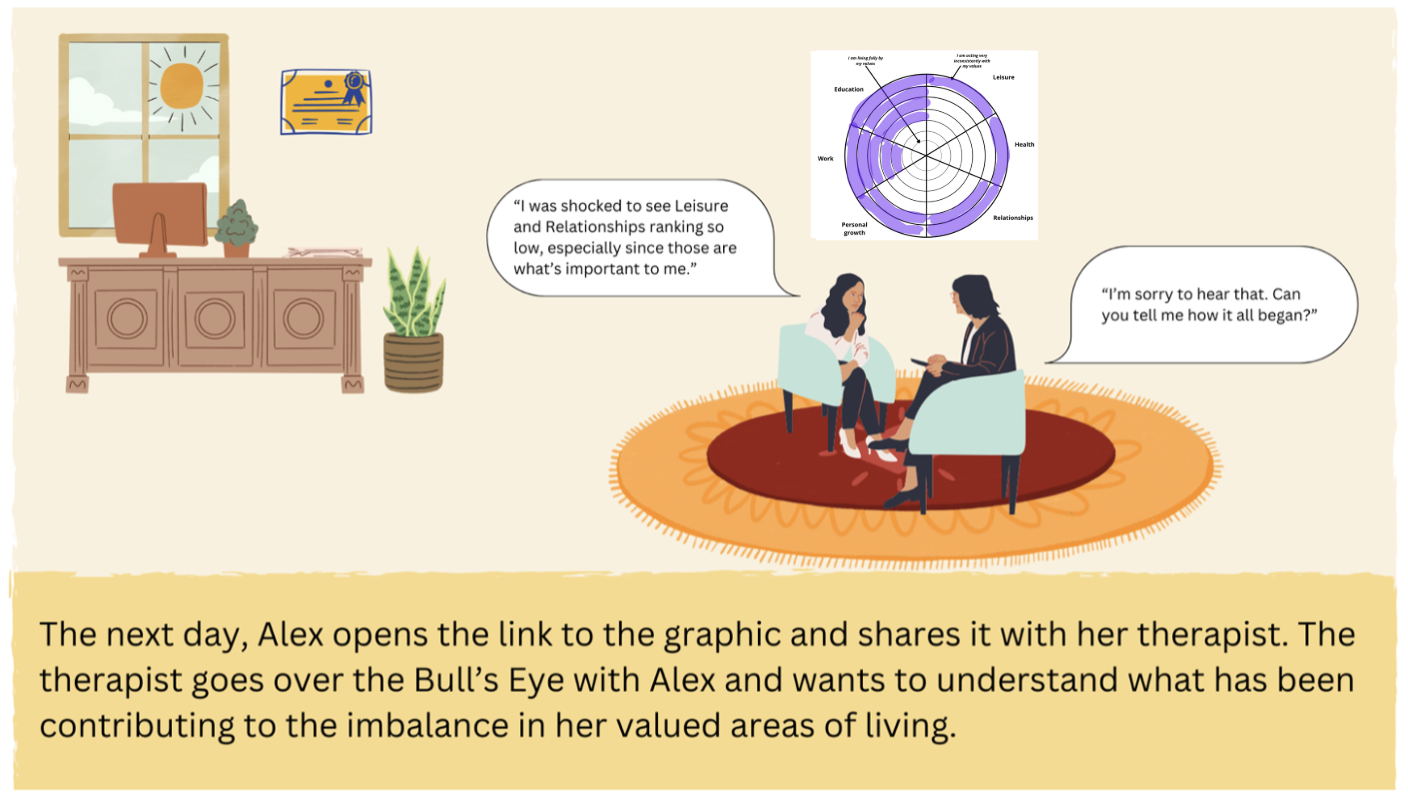


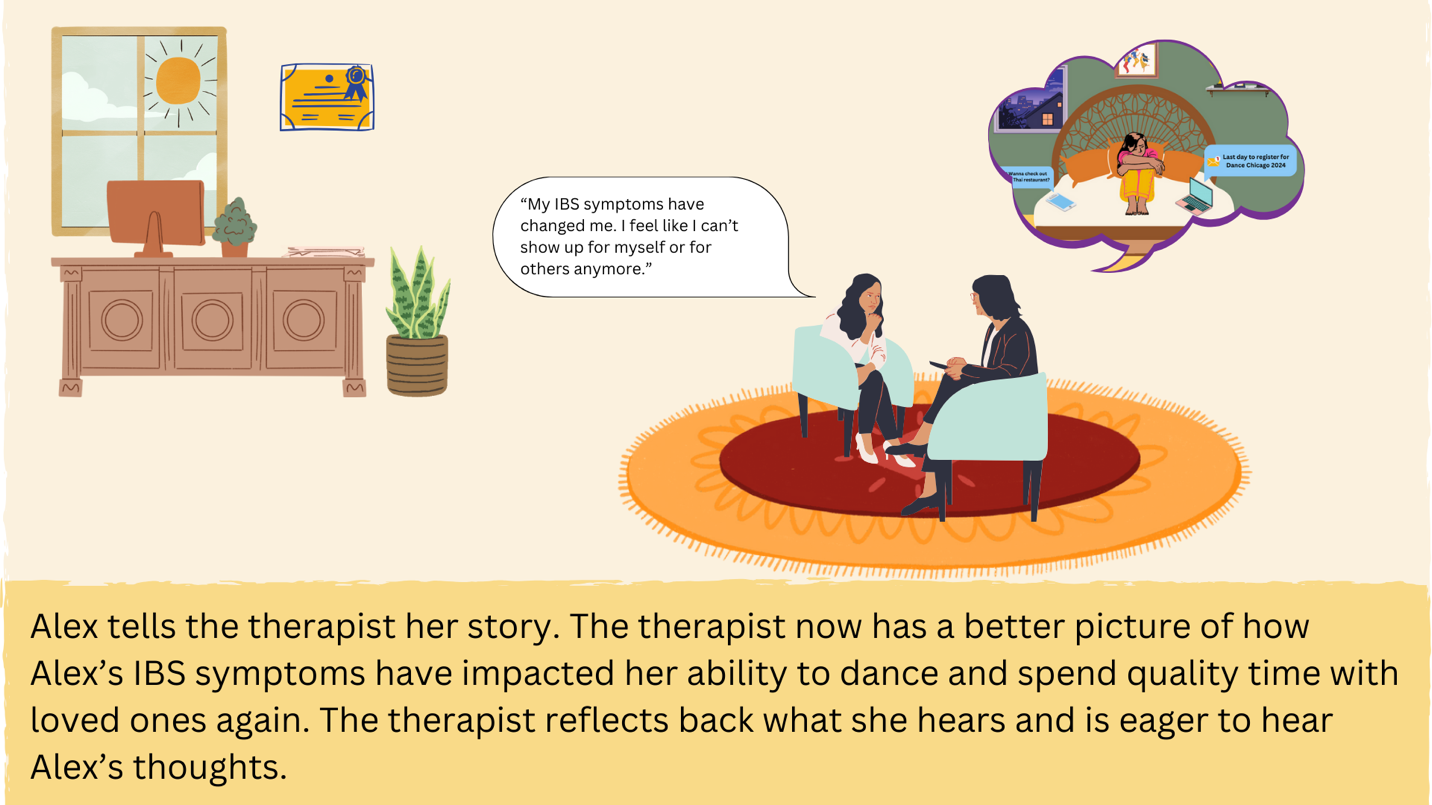


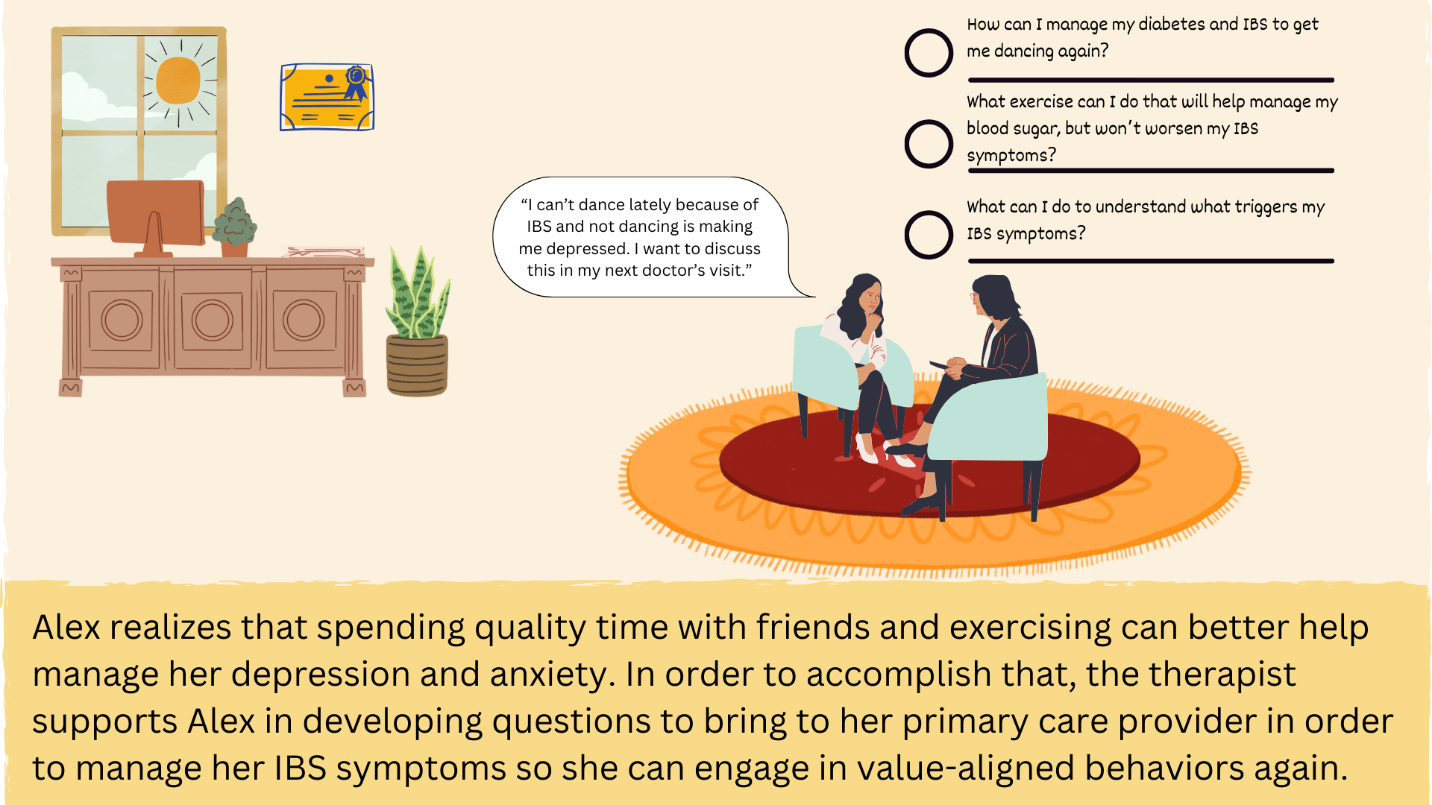


**Multimedia Appendix 1G.** “PCP Simulator” (Workshops 6-7). Pre-appointment, patients use a chatbot to practice communicating with their PCP and receive takeaways including communication tips and appointment priorities.


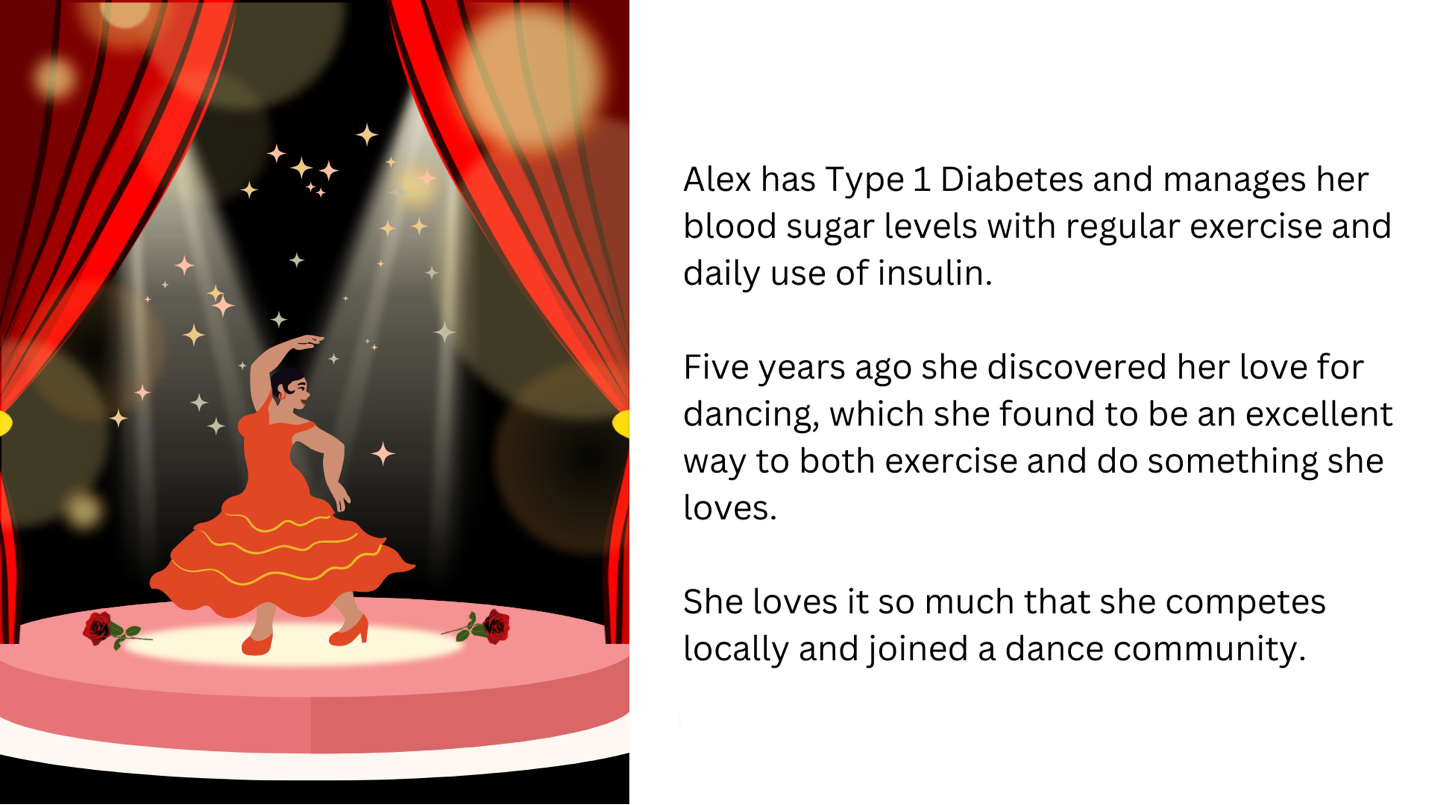


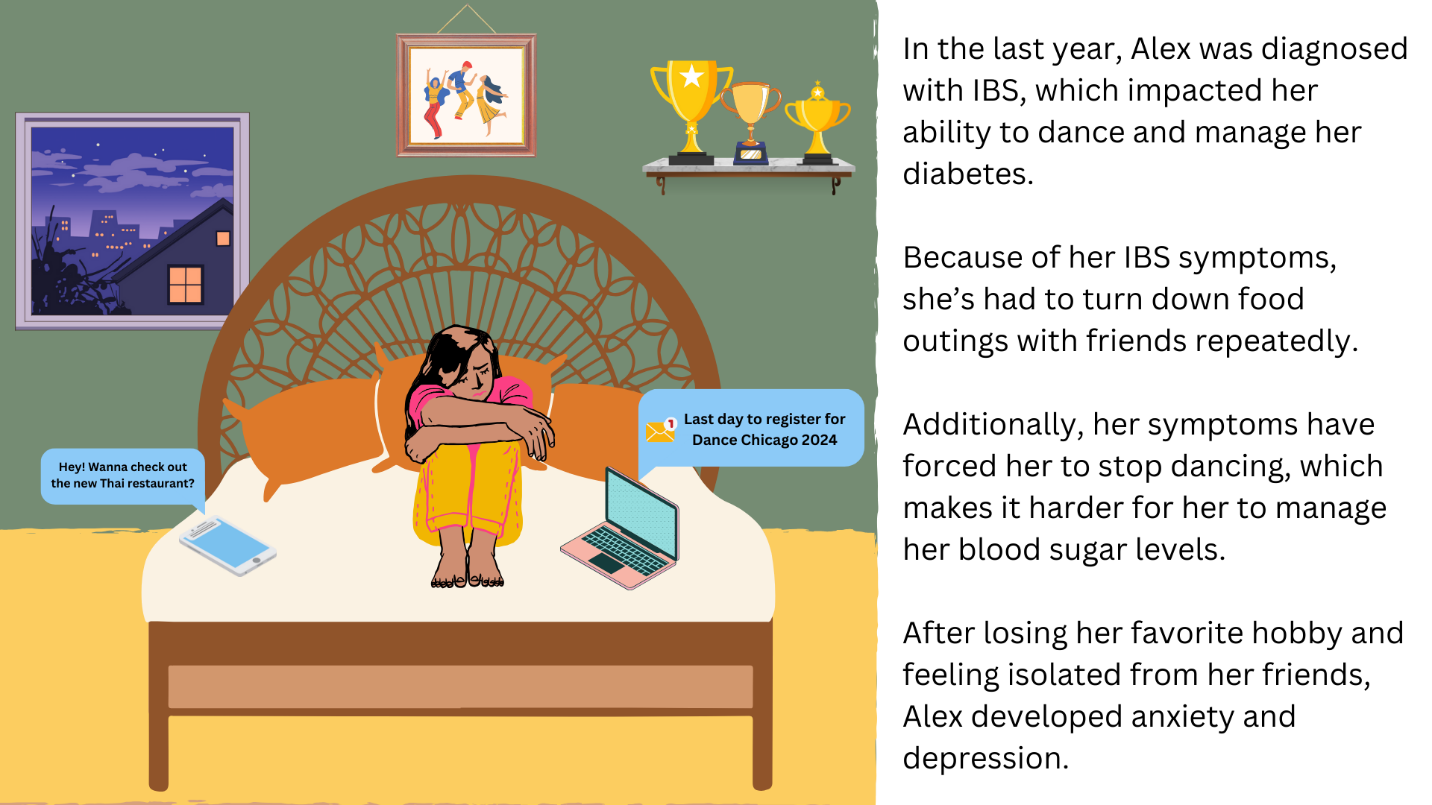


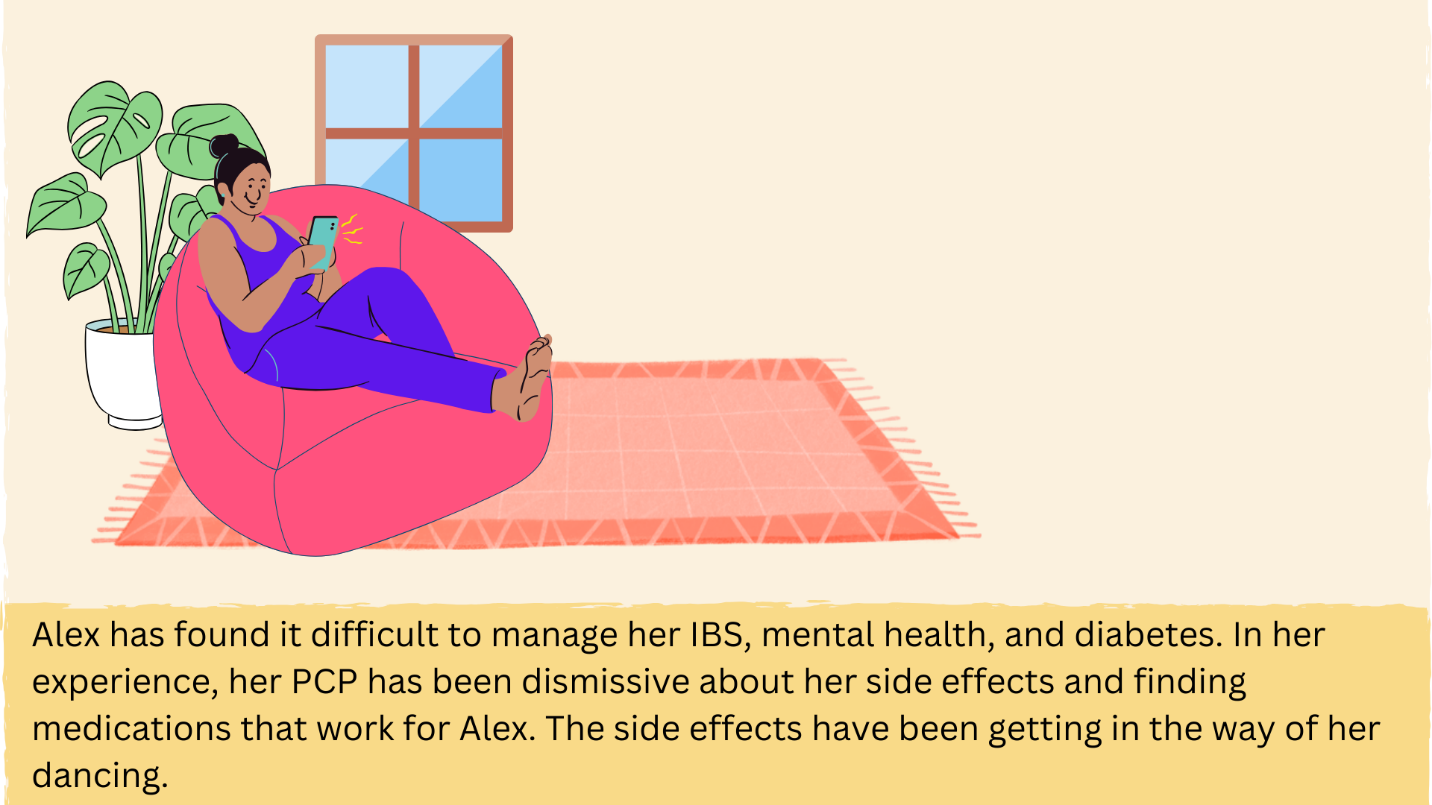


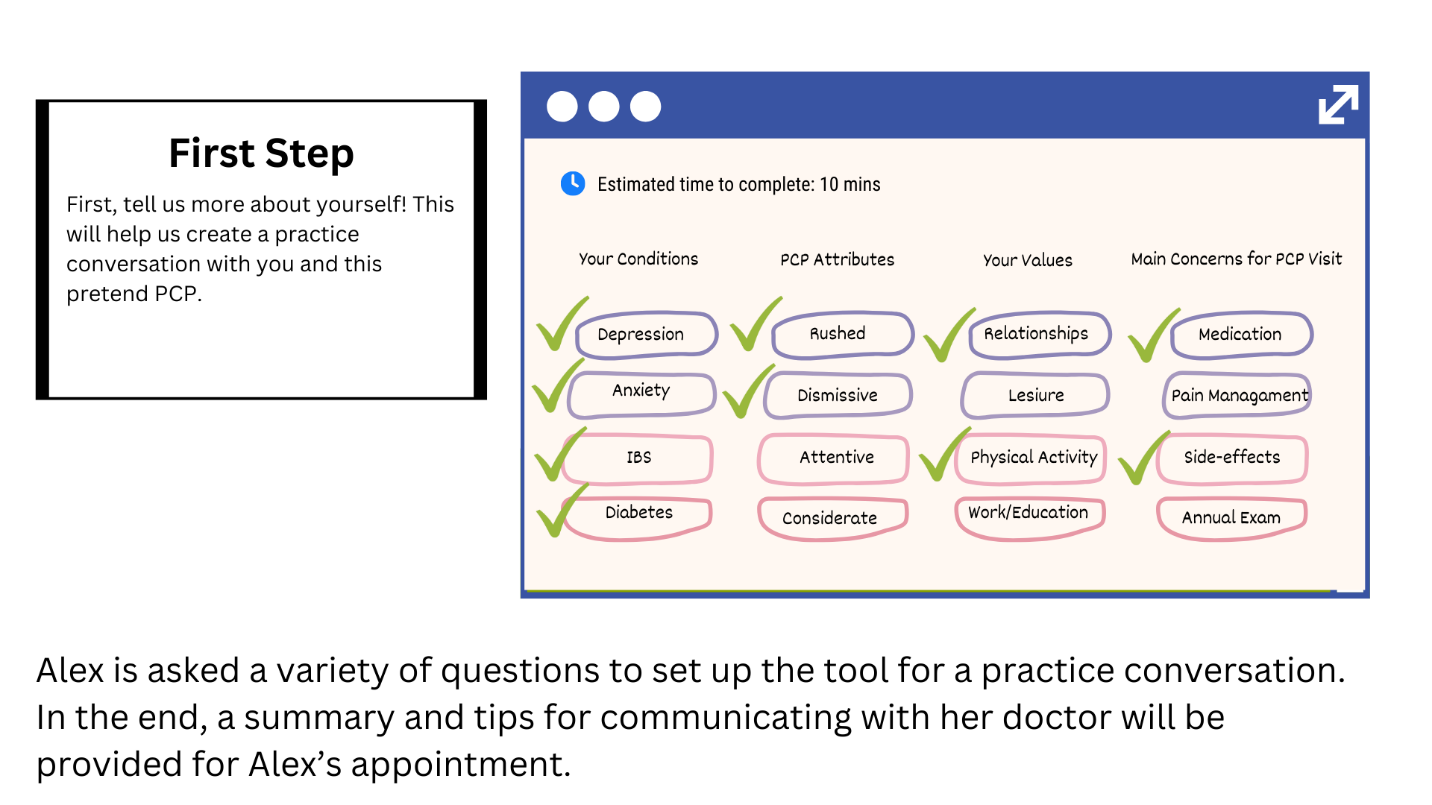

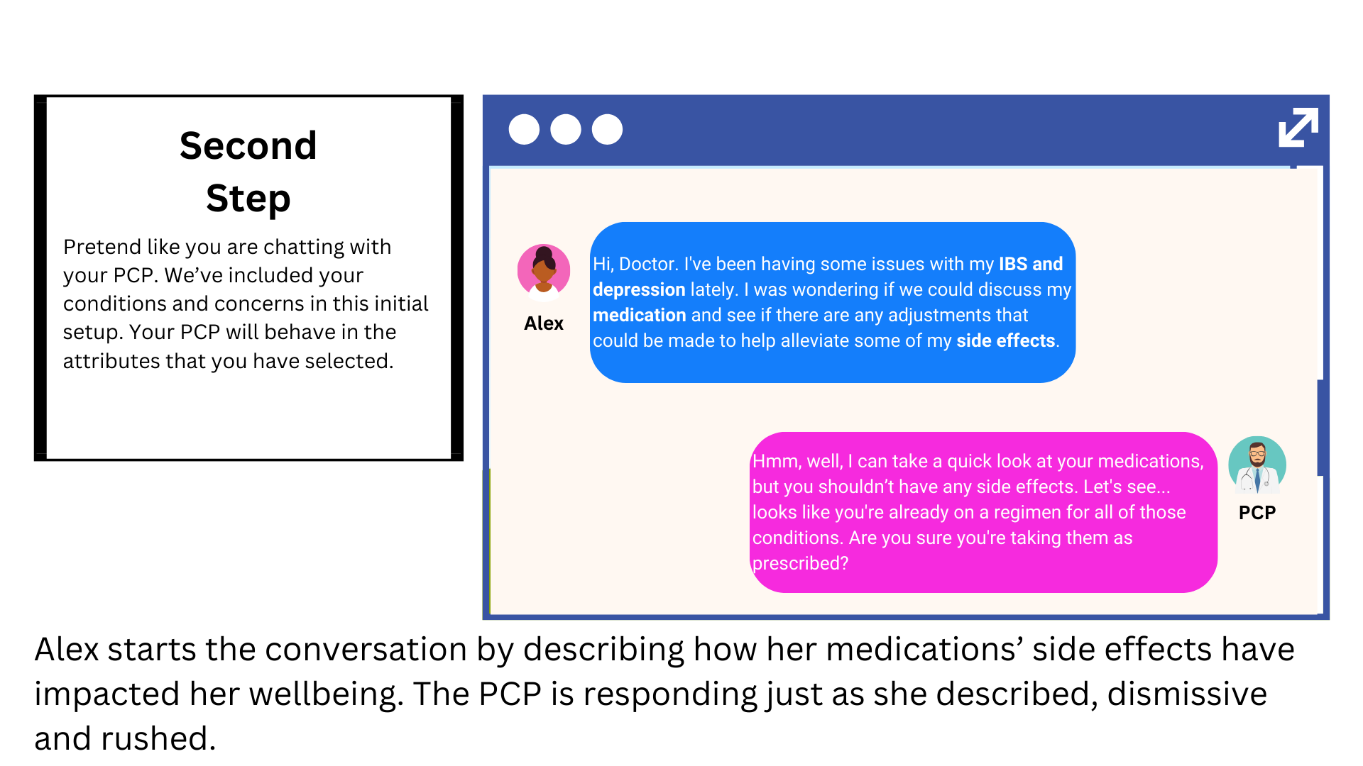

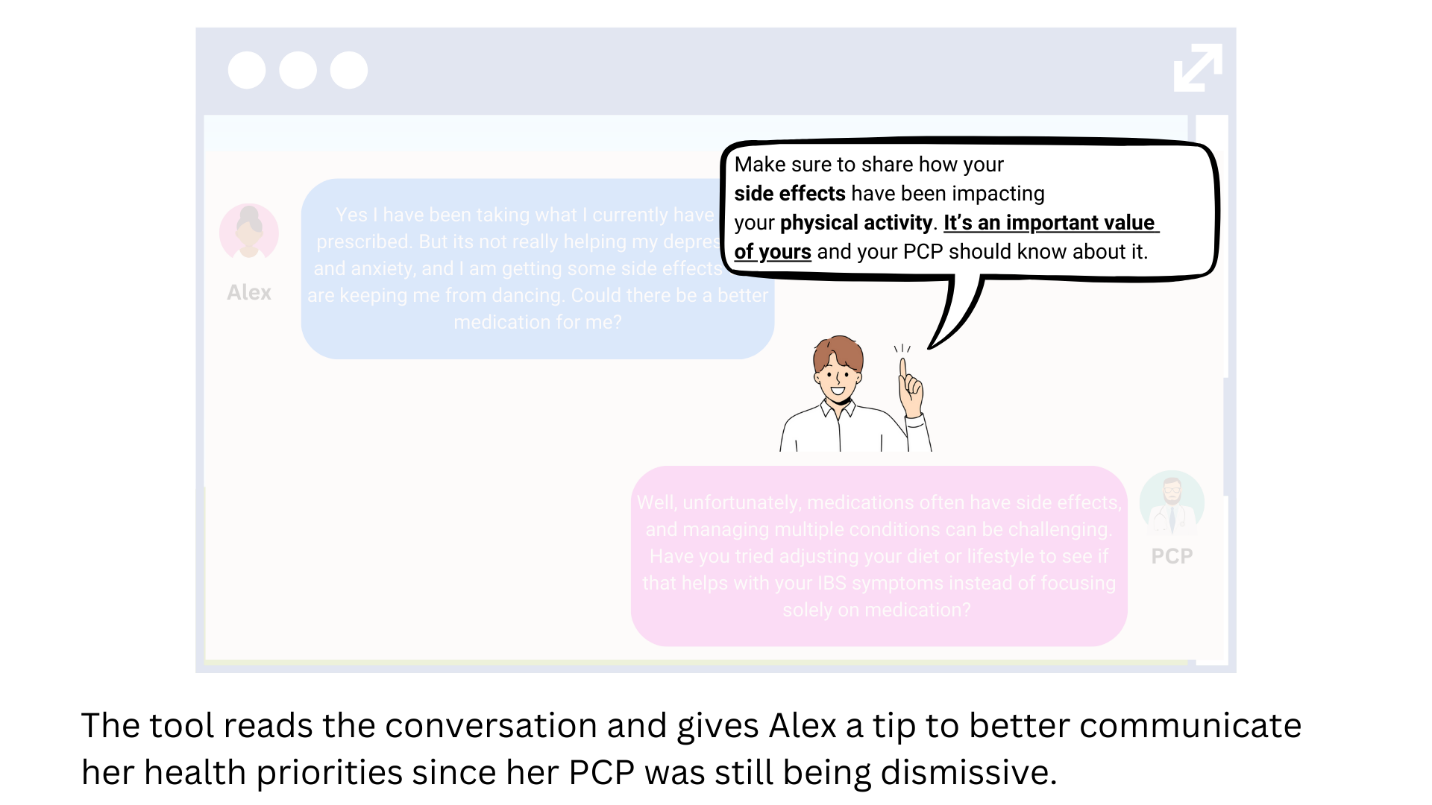

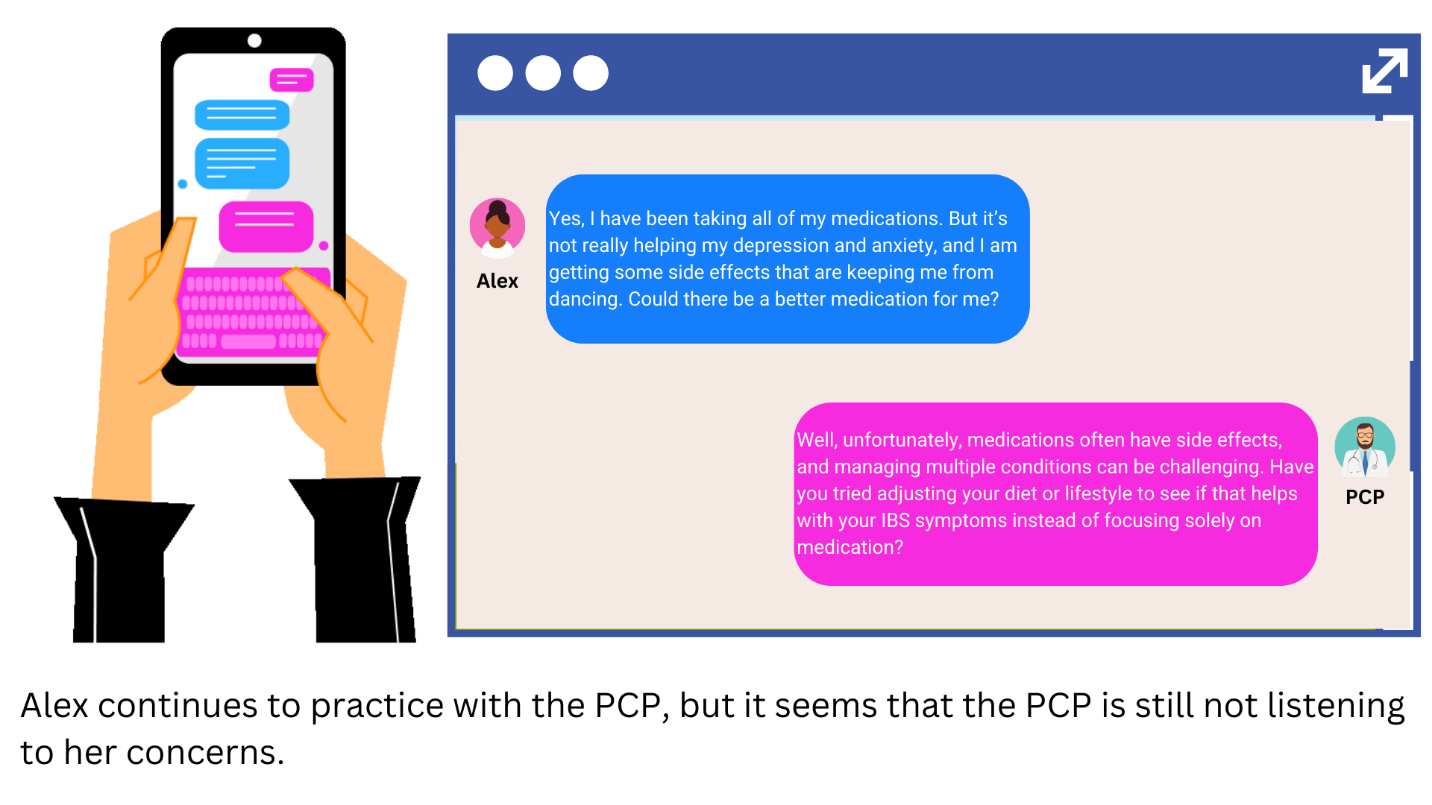


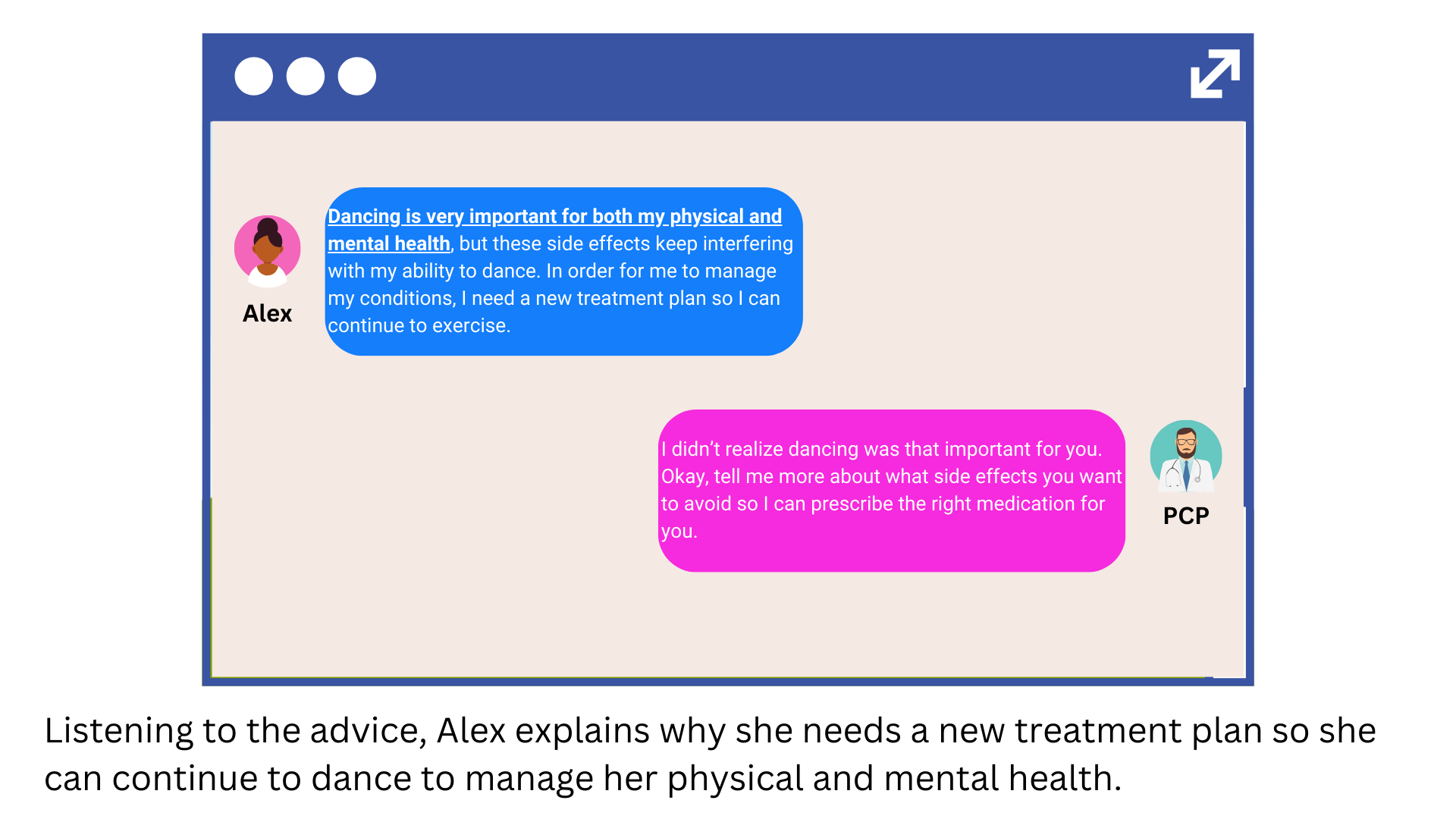

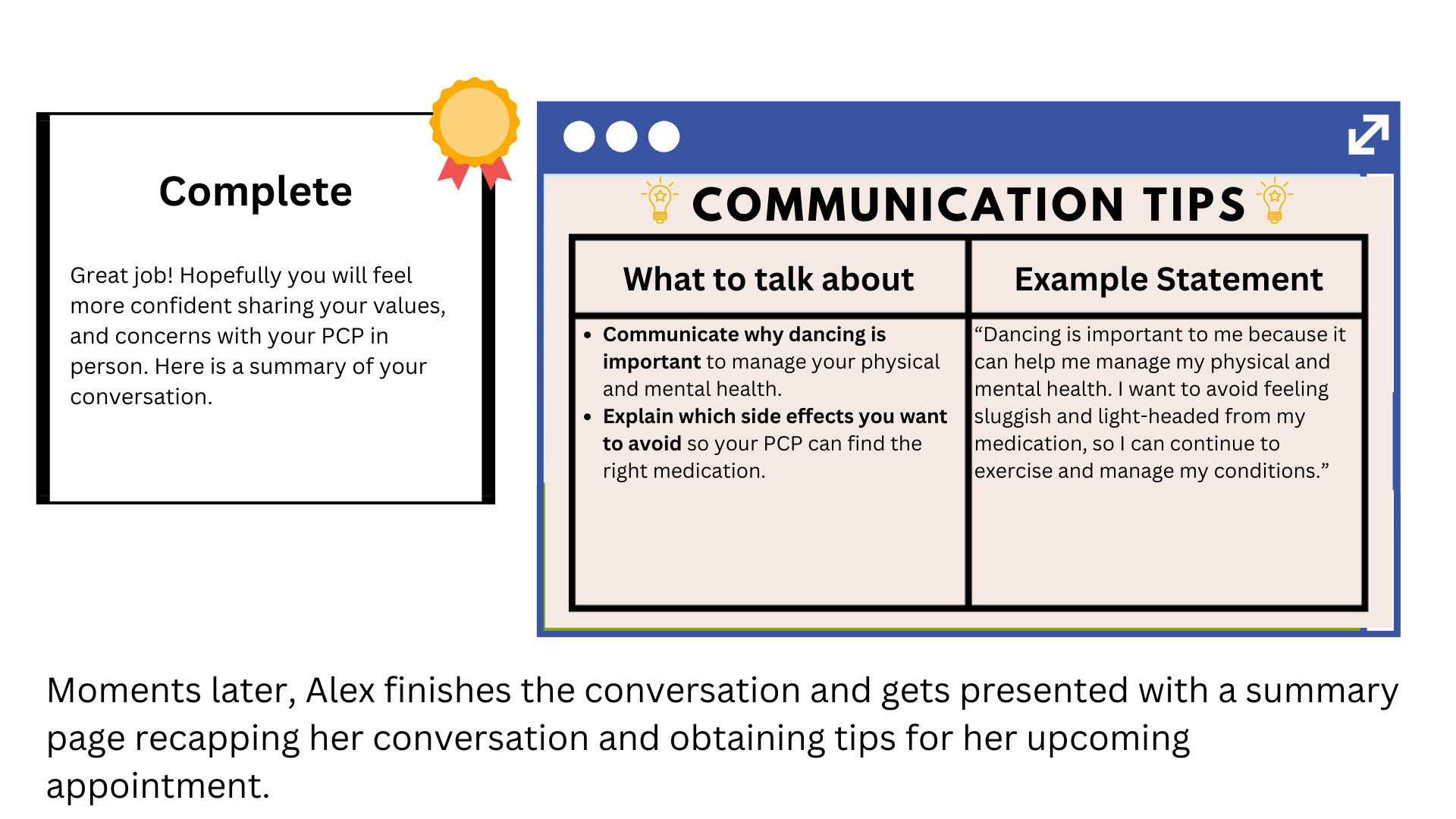

Supplement: Multimedia Appendix 1 [file formative_v9i1e68419_app1.docx]
